# Mindfulness's moderating role applied on online SEL education

**DOI:** 10.3389/fpsyg.2024.1499357

**Published:** 2024-11-19

**Authors:** Chun-Heng Ho, Hang-qin Zhang, Juan Li, An'an Liu

**Affiliations:** ^1^Department of Industrial Design, College of Planning and Design, National Cheng Kung University, Tainan, Taiwan; ^2^Department of Industrial Design, College of Mechanical Engineering and Automation, Huaqiao University, Xiamen, China; ^3^Department of Compose, Fontys University of Applied Sciences, Tilburg, Netherlands

**Keywords:** emotional problem, digital education, teaching effect, emotion management, music therapy

## Abstract

**Introduction:**

Mild to moderate depression, anxiety, and stress imbalances are prevalent emotional issues among college students and are primary factors leading to deficiencies in social-emotional skills within this population. Without timely intervention, these mild to moderate emotional issues may escalate into more severe conditions. Social-Emotional Learning (SEL) programs are effective for building social-emotional skills. However, current research on SEL programs has not adequately addressed the issue of high-quality teacher-student interactions for students who suffer emotional problems. To tackle this issue, this study proposes a curriculum approach that integrates mindfulness with rhythmic music? and evaluated the emotional changes of students after mindfulness with rhythmic music curriculum.

**Methods:**

This study adopted a pre-post experimental design. Two hundred and ninety-four firefighting universities students participated in a one-semester “online mindfulness combined with music rhythm SEL course”. The study used the Beck Anxiety Inventory, Center for Epidemiologic Studies Depression Scale and Perceived Stress Scale to measure the anxiety, depression and stress levels of the participants before and after the course, and used the participants' self-reflection reports as a method to explore the students' emotional transformation patterns.

**Results:**

The research findings indicate that: (1) eighth-note, quarter-note, and sixteenth-note rhythmic music significantly improve the emotional wellbeing of students with depression, anxiety, and stress imbalances, respectively. (2) The degree of emotional improvement has a certain impact on academic performance. (3) Students with anxiety require more instructional support focused on attention concentration during the early phases of the course; students with depression should not be scheduled for social skills learning modules in the short term and need long-term instructional guidance; individuals experiencing stress imbalances require attention to their personal music preferences and benefit from additional listening activities and exercise.

**Discussion:**

These findings assist teachers in accurately identifying emotional changes among students with emotional problems and managing the patterns of these emotional transitions, thereby providing effective instructional support and promoting high-quality interactions between teachers and students.

## 1 Introduction

Driven by digitalization, online social-emotional learning (SEL) programs have increasingly become a vital pathway for fostering personal growth and sustainable educational development in students. These programs encompass not only the abilities related to emotional recognition and expression but also involve skills in decision-making, goal setting and achievement, as well as managing stress and challenges. Numerous studies confirmed that SEL programs contribute to improving students' emotional health, enhancing life satisfaction, and fostering better interpersonal relationships (Jones et al., 2017). Additionally, SEL significantly impacts academic achievement, teacher-student relationships, and classroom atmosphere (Poulou, 2014; Lawlor, 2014; Schonert-Reichl et al., 2015; Lawlor, 2016; Conley, 2015). Consequently, countries around the world are vigorously promoting social-emotional learning (SEL) programs. For instance, as early as 1982, the United States introduced social skills curricula for selected schools with antisocial behavior children (Van Andel, 1982). Subsequently, the American Institutes for Research (AIR) implemented SEL in selected districts nationwide through the Collaborative District Initiative (CASEL), which demonstrated both the successes and challenges of implementing SEL district-wide (Kendziora and Osher, 2016). In the United Kingdom, localized social skills/emotional skills curricula were provided for at-risk students in 1998, organized jointly by local education authorities and educational psychology services in London, Birmingham, Manchester, and Lancaster. Two years later, the University of Southampton established the Southampton Emotional Literacy Interest Group (SELIG) and held the first Emotional Literacy Schools Conference in 2002 (Emery, 2016). Australia has developed extensive SEL resources, such as KidsMatter, an integrated online resource designed in collaboration with mental health service providers aimed at schools, early childhood educators, families, and community workers. This initiative provides SEL resources to many indigenous schools in Australia and has established programs like MindMatters, a secondary school-based mental health initiative (Australian Goverment Department of Health, 2015). In Poland, the Institute of Psychology at Adam Mickiewicz University in Poznań collaborated with the Greater Poland Province School Inspectorate to launch a SEL outreach initiative in 2013 (Bowles et al., 2017). In Portugal, three school groups collaborated to implement a secondary school SEL program for middle school students. Follow-up evaluations indicated that most students benefited positively from the program, supporting the effectiveness of SEL initiatives (Coelho et al., 2015). Singapore has established SEL as a guiding principle for cultivating social-emotional competencies (SEC) among its students. The Ministry of Education conducted an international evaluation of best SEL practices from 26 projects and 20 frameworks across several countries, including Australia, China, South Korea, the United States, and the United Kingdom, which was used to comprehensively promote SEL in schools. Singaporean schools have integrated SEL as a primary preventive intervention across all levels of education (Chong and Lee, 2015; Liem et al., 2017). Thousands of schools in the United States, the United Kingdom, Australia, Portugal, Poland, Singapore, and other countries have incorporated social-emotional learning into their curriculum design, aiming to enhance students' social and emotional capabilities and improve educational quality.

Despite the widespread implementation of Social-Emotional Learning (SEL) programs and the positive results achieved in many cases, some studies have pointed out that discrepancies in the quality of SEL program implementation have led to uneven results (Coelho and Sousa, 2018; Domitrovich et al., 2019; Dowling and Barry, 2020). Poor quality of SEL outcomes has resulted in decreased trust in their efficacy among certain groups or institutions, such as teachers, schools, and relevant functional departments, significantly hindering the comprehensive and in-depth promotion of SEL across society and causing a stagnation in the exploration and development of social-emotional skills. Consequently, the issue of subpar SEL program. Extensive research has sought to identify the reasons for enhancing the quality of SEL program implementation. Many studies explore the factors affecting the final quality of outcomes in the process from the development to the implementation of SEL programs across multiple dimensions, including the SEL program framework and measures, teacher training, school support, and implementation quality assessment, while also proposing relevant solutions. For example, to address the lack of attention in the SEL program framework to different groups (such as age, race, cultural background, family environment, and income), some researchers advocate for culturally integrated SEL skill development, project-based learning (PBL), and youth participatory action research (YPAR) (Umaña-Taylor et al., 2017; Bui and Fagan, 2013). Other studies suggest establishing pre-service teacher education programs to help teachers gain a deeper understanding of SEL initiatives and to address the issue of teachers lacking pre-service training in SEL programs (Sandilos et al., 2023; Murano et al., 2019). Some research emphasizes increasing oversight by schools and relevant legislative bodies regarding SEL programs to promote school-level accountability for social and emotional curricula (Elbertson et al., 2019). Additionally, certain studies have developed multidimensional frameworks for evaluating SEL programs, enabling more comprehensive and systematic monitoring of the quality of SEL implementation. This approach helps to identify the factors that contribute to poor implementation quality, facilitating the formulation of strategies to optimize the quality of SEL interventions (Durlak and DuPre, 2008; Domitrovich et al., 2008; Humphrey et al., 2018). These case studies demonstrate a rich array of solutions proposed by numerous researchers to address the issue of inadequate implementation quality in SEL programs. Although these innovative suggestions have achieved certain positive outcomes, they tend to focus on the impact of single dimensions on final outcome quality. However, implementation quality is a complex process that depends on many interacting dimensions (Proctor et al., 2009). Currently, there is limited research that combines multiple dimensions to explore how their interactions affect final outcomes. Notably, the quality of the interaction among students, teachers, and the classroom is often overlooked, even though SEL skills and behaviors are formed within these relationships. Currently, there is limited research that combines multiple dimensions to explore how their interactions affect final outcomes. Notably, the quality of the interaction among students, teachers, and the classroom is often overlooked, even though SEL skills and behaviors are formed within these relationships.

Growing evidence suggests that high-quality teacher-student classroom interactions play a crucial role in SEL learning (Poulou, 2014; Poling et al., 2022). This significance arises from the fact that SEL programs are often delivered either as standalone courses or integrated into regular curricula, making the implementation process fundamentally a teaching activity. Moreover, SEL instruction tends to establish a student-centered classroom, prioritizing students' interests and emphasizing concepts such as prioritizing student discussion over teacher lecturing, strategic thinking to solve problems, and providing opportunities for students to learn from their mistakes (Boston Office of Education, 2017). This approach aims to facilitate students' internal self-exploration. Overall, the educational philosophy and implementation form of SEL dictate that its essence lies in the teaching process, which necessitates close interaction among students, teachers, and the classroom to achieve high-quality outcomes. Therefore, high-quality teacher-student interactions are a critical factor influencing the quality of SEL outcomes. Additionally, these interactions create a positive feedback loop for subsequent teaching and learning within SEL programs. For students, effective and deep teacher-student interactions foster engagement, participation, and responsiveness, leading to a profound understanding of the learning material and positive academic outcomes. This, in turn, enhances students' self-confidence and better equips them to establish long-term emotional and social skills, laying a solid foundation for their ongoing sustainable learning. For teachers, deep interactions with students are built on a thorough understanding of their students, enabling teachers to timely develop and adjust instructional plans, summarize students' learning patterns, and provide a theoretical and practical groundwork for future curriculum improvements and pedagogical innovations. This cyclical process ultimately establishes the foundation for the formation of a sustainable, high-quality curriculum across generations.

Research evidence indicates that emotional support (characterized by positive emotions, warmth, and responsiveness; awareness of students' needs), classroom organization (effective use of teaching methods, fostering student motivation and engagement, and ensuring student compliance), and instructional support (personalized feedback, scaffolding, enriched language exposure, and activities that promote higher-order thinking) are crucial components of deep, high-quality teacher-student interactions (Hamre and Pianta, 2003; Pianta et al., 2015). High-quality teacher-student interactions necessitate a close integration of these three aspects to create an emotionally supportive classroom environment. Specifically, it is essential for teachers to be sensitive to students' needs and interests while employing various strategies to enhance classroom organization and provide supportive instructional interactions, which are crucial for promoting classroom learning (Hamre and Pianta, 2010). However, existing research has not sufficiently equipped teachers to understand and master this interaction process, particularly failing to address the important issue of how teachers should perceive changes in students' emotions to provide effective instructional support at appropriate times. This challenge is particularly pronounced for students with existing mild to moderate emotional problems, as their interactions and communications with teachers may be more difficult. Therefore, establishing effective instructional support for students with emotional issues presents a significant challenge.

### 1.1 Importance of addressing teacher-student interaction challenges for students with emotional issues in SEL programs

Emotional issues have become a serious and frequently occurring problem among students, particularly within the college student demographic. Three specific emotional problems—depression, anxiety, and stress imbalances—are particularly prevalent among college students, with reported prevalence rates of 75, 88.4, and 84.4%, respectively (Asif et al., 2020). One study found that 13% of students can be categorized as “vulnerable”, those experiencing mild to moderate emotional issues. These students exhibit lower levels of emotional wellbeing and subjective happiness. Compared to emotionally healthy students (defined as those with low emotional problems and high subjective wellbeing) vulnerable students demonstrate weakened academic self-concept, a more negative perception of the importance of school, decreased motivation for self-regulatory behaviors necessary for learning (Green et al., 2021), and behaviors characterized by social avoidance, withdrawal, and isolation. This results in negative impacts on their self-image and interpersonal relationships, exacerbating issues related to introversion, low self-esteem, or sensitivity, which further hinder the establishment and maintenance of relationships (Chiou et al., 2021; Chen et al., 2023). These manifestations reflect the difficulties that students with emotional health issues face in clearly expressing their viewpoints and thoughts to teachers, leading to teachers' inability to accurately assess students' emotional changes and learning progress. This creates a barrier to establishing a positive and healthy interaction between students and teachers. This is why regular students may achieve positive outcomes while those with emotional issues continue to struggle. If left unaddressed, early emotional health problems in students may lead to long-term adverse consequences. For students, mild emotional health issues are highly likely to develop into severe emotional problems, potentially triggering a series of somatic symptoms. Consequently, these students may fall behind their peers, experience decreased engagement in learning, lose enthusiasm and interest in education, and exhibit behaviors that contribute to school avoidance and dropout. Ultimately, this loss of connection with peers, school, and community can plunge students deeper into a vortex of negative emotions, further exacerbating emotional problems, and in severe cases, lead to extreme behaviors such as self-harm or suicidal tendencies. From a school perspective, the prolonged and widespread emotional malaise among students inevitably disrupts normal teaching activities and has detrimental effects on the school's future development. Furthermore, when these students enter society, the long-standing emotional issues that have not been alleviated or treated make them more susceptible to criminal behaviors, educational challenges, and unemployment issues, severely impacting the smooth functioning and sustainable development of society. Over time, this creates a vicious cycle that ultimately affects social cohesion and stability. Therefore, addressing the difficulties in deep teacher-student interactions within SEL programs for students with emotional health issues and aiding in the establishment of their social-emotional skills is an urgent and critical issue.

Although current SEL research has begun to address this specific group, studies specifically targeting students with emotional issues remain scarce. Mainstream interventions tend to focus on emotional expression. However, emotional expression itself does not necessarily promote positive mental health, and acceptance of emotional experiences and expression varies across cultural groups (Sue and Sue, 2013). This is particularly true for students with emotional health issues, some of whom are resistant to establishing interpersonal relationships. Furthermore, methods of emotional expression may not be universally applicable across different cultural contexts, with notable differences between Western and East Asian societies. Individuals from Asian cultures, who may be more adept at suppressing emotions, report fewer negative feelings and may regulate and cope with these emotions in different ways, such as through somatization (Kleinman, 1986). Lee (2013) indicates that among students in Singapore, those experiencing somatic complaints like headaches, weakness, or lack of energy may not be suffering from disorders indicative of psychopathology, but their symptoms may actually reflect culturally specific emotional expressions (Kirmayer and Sartorius, 2007). In societies where emotional expression is regarded as a conventional cultural norm, individuals may feel significant pressure not to share their feelings when they choose not to (Lee, 2013; Quinton and Wagner, 2005). In such circumstances, students with emotional problems may face considerable limitations in expressing themselves due to the characteristics of their conditions, leading to obstacles in normal communication between students and teachers. This hinders teachers' ability to provide effective support for students' SEL learning. Therefore, there is an urgent need for teachers to adopt methods that do not require students to express their emotional feelings but can help them identify the emotional experiences, levels of emotional change, and temporal differences among various groups of students with emotional problems. Such methods would enable teachers to manage the patterns of emotional transitions among different student populations, thus providing scientific references and methodologies for effective instructional support for students with emotional issues. Ultimately, this would enhance the quality of teacher-student interactions for students with emotional health problems.

In summary, this study proposes the implementation method of a social-emotional learning (SEL) program that integrates mindfulness with rhythmic music. The aim is to assist teachers in accurately identifying the emotional experiences and emotional transitions of students facing three types of emotional issues: depression, anxiety, and stress imbalances. By managing the patterns of emotional transitions among these student populations, teachers can provide more effective instructional support for students with emotional problems, thereby promoting deep interactions between teachers and students and enhancing the learning outcomes of SEL programs among college students experiencing high rates of depression, anxiety, and stress imbalances. Additionally, this approach provides a scientific and objective foundation for the sustainable improvement and implementation of future SEL programs. The following sections will explain the characteristics of this program method in detail.

## 2 Methodology

### 2.1 Mindfulness

Various approaches have been adopted in existing SEL programs, such as the Promoting Alternative Thinking Strategies (PATHS) (Kusché and Greenberg, 2012), the Child Development Project (CDP) (Battistich et al., 1991), and Classroom Emotional Literacy (ELC) (Kremenitzer et al., 2008), as well as Culturally Relevant Education (CRE), Project-Based Learning (PBL), and Youth Participatory Action Research (YPAR) (Vasquez, 2015; Holthuis et al., 2018; Ozer, 2016). These methods generally focus on helping students recognize emotions, such as collaboratively completing emotion-naming exercises or expressing their emotional feelings. For example, students might be invited to mark different emotional categories on a poster and place name stickers to indicate their current emotional state. However, such methods can be challenging for students who are not comfortable with or are unwilling to express emotions publicly. Mindfulness serves as an effective external emotion regulation method and can also become an internalized emotional regulation capacity (Hayes and Feldman, 2004). Mindfulness training focuses on maintaining awareness of a particular object (such as breathing, external stimuli, thoughts, bodily sensations, etc.) to train attention, monitor the wandering state of thoughts, and, upon realizing that thoughts have wandered, gently return focus to the chosen object. The process of mindfulness training helps students become aware of their consciousness and what is happening in their surroundings without judgment, analysis, or reaction (Liu et al., 2020). This aligns with the previously mentioned requirement of helping students recognize emotions without mandating emotional expression. Moreover, mindfulness has been widely applied in various emotional problem interventions and SEL programs, yielding positive results (Liu et al., 2021; Chiou et al., 2022; Flook et al., 2024; Liu et al., 2023). For instance, among nine reviewed mindfulness interventions, seven (78%) were successful in improving emotional distress, self-awareness, and social-emotional skills post-intervention. However, when mindfulness interventions are implemented in isolation or as the sole strategy, they can aid students in recognizing and regulating emotions but may not provide sufficiently precise support tailored to the varying needs of different students with emotional problems. This makes it challenging for teachers to identify students' emotional transitions and clarify individual differences in their emotional changes. Consequently, there needs for a method that offers specific alleviation effects tailored to different emotional issues. Increasing evidence suggests that music therapy can effectively alleviate symptoms in populations with emotional health problems (Ward, 2022; Varner, 2023; Çenberci and Tufan, 2023). Additionally, psychological research indicates that the combination of mindfulness and music is considered an effective means of providing personalized support. Specifically, music has the ability to evoke emotions, and listening to music while in a mindful state can help learners better accept their emotions. For example, Lesiuk (2015) demonstrated that an intervention combining mindfulness and music significantly improved attention in female patients with breast cancer. Importantly, some studies have revealed that rhythmic music can provide targeted assistance for various emotional health problems.

### 2.2 Music rhythm

Musical rhythm is composed of vibrations of different frequencies, which facilitate the transmission of mechanical energy and form the dynamic basis of music. Its structure, along with its dynamic and predictable nature, can be received by various parts of the body, producing resonance. The amplitude and frequency of vibrational stimuli can elicit psychophysiological responses (Watkins, 1997). The rhythms can effectively support mindfulness practices; for instance, faster-paced music can enhance attention and boost energy, maximizing the effects of mindfulness. Moreover, varying rhythms within a piece of music can address different emotional health needs, which is particularly beneficial for students with emotional issues (Wu, 2023). Research has shown that fast-paced music combined with exercise significantly improves depression. A study comparing the effects of rhythmic vs. non-rhythmic aerobic exercise on depression in older adults utilized music rhythms ranging from 125 beats per minute (bpm) to 160 bpm during aerobic exercise. The results indicated that the combination of musical rhythm and exercise had a significant positive effect on alleviating depression among older adults (Kwon et al., 2017). Furthermore, relaxing rhythmic music, such as 4/4 or 8/8 time, has been shown to effectively control anxiety. This is attributed to the simplicity, consistency, and relative subtlety of these rhythms, which can significantly aid relaxation and alleviate anxiety (Elliott et al., 2011). Numerous studies have confirmed that music can effectively reduce stress (Bokiev et al., 2018; Joshi and Kiran, 2020; Feng, 2022). One study indicated that music activities and rhythm-based stress perception training could alleviate students' psychological stress. In summary, musical rhythm therapy may effectively address the limitations of implementing mindfulness therapy in isolation, particularly in providing targeted assistance to different groups with emotional issues. This approach can help teachers accurately identify the emotional transitions of students with varying emotional problems, clarify group differences, and manage the patterns of emotional transitions among students. Consequently, this will enable teachers to offer instructional support that aligns with students' needs, ultimately enhancing deep interactions between teachers and students and improving the quality of SEL program implementation across different populations.

### 2.3 Research objectives

The primary objective of this study is to investigate which musical rhythms in a mindfulness-based SEL program provide targeted and effective alleviation of emotional problems related to depression, anxiety, and stress imbalances among students, thereby improving their academic performance. Additionally, we aim to explore the patterns of emotional transition points for students experiencing emotional issues such as depression, anxiety, and stress imbalances while participating in the SEL program. This knowledge will assist teachers in managing the emotional transitions of different student populations with emotional problems and provide instructional support to enhance deep interactions between teachers and students, while also expanding the applicability of the mindfulness-based music rhythm curriculum as much as possible. To achieve this, we conducted an experiment that compared the emotional health status and academic performance of students in different musical rhythm groups before and after participating in the SEL program, as well as their emotional transitions during the learning process.

## 3 Development of the mindfulness-integrated music rhythm program

The mindfulness combined with rhythmic music curriculum (hereafter referred to as the “new curriculum”) integrates theories and research from mindfulness, social-emotional learning (SEL), and music science. The curriculum was collaboratively developed and organized by a team comprising educators who have participated in mindfulness and SEL training, as well as teachers with years of composition experience. With the support of partnerships with schools and communities, the research team worked with teachers, school personnel, and community workers prior to the academic year to identify the areas of need they wished to address through SEL, including alleviating student emotional issues, improving academic performance, and enhancing wellbeing. The curriculum is structured around the five competencies originally established by CASEL: self-management, self-awareness, social awareness, relationship skills, and responsible decision-making. Each course plan focuses on one competency, consisting of one lesson and a self-reflection activity. The overall curriculum is divided into three chapters comprising ten lessons, focusing on self-awareness, social awareness, and responsible decision-making. The self-awareness chapter includes three sections: recognizing emotions, emotional awareness, and emotional regulation. Its primary goal is to cultivate students' ability to identify their own emotions and thoughts as well as to regulate their emotional states. This chapter primarily employs mindfulness techniques, particularly breath-focused exercises, to help students reduce stress, enhance concentration, and improve mood. The social awareness chapter consists of five sections: perspective-taking, empathy for others, anger management, conflict management, and conflict resolution. This chapter aims to foster students' appreciation for diversity and differences, respect for others, listening skills, sensitivity, and empathy toward the feelings of others, thereby helping them build their social skills and laying the foundation for positive social interactions in the future. Additionally, this chapter focuses on developing interpersonal relationship skills, such as conflict resolution, aimed at equipping students with the ability to establish and maintain healthy, reciprocal relationships with individuals from diverse social and cultural backgrounds, resist negative social pressures, manage conflicts, help others, and seek help. This chapter incorporates activities such as active listening, reflection, and mindfulness meditation to aid students in learning how to manage their emotions. The responsible decision-making chapter includes sections on making positive life choices and making choices based on awareness and compassion. Its primary goal is to provide students with a practical platform to develop their ability to make wise, ethical, and effective choices in interpersonal and social interactions. This chapter employs methods such as mindfulness activities and community service learning, encouraging students to participate in social action projects to perform acts of kindness, such as community service, charity events, and volunteering, thereby helping them enhance their practical skills.

Additionally, the new program incorporated three different musical rhythms to complement mindfulness practices: quarter-note, eighth-note, and sixteenth-note rhythms. During mindfulness exercises, students listened to one of these rhythms based on their specific emotional challenges. The choice of rhythm was tailored to the classification of emotional issues, aiming to enhance the effectiveness of the mindfulness sessions. The specific course units and plans are outlined in Table 1.

**Table 1:** Units of the mindfulness-based music rhythm curriculum.

**Unit**	**Key focus**	**Mindfulness practices**	**Music**
Self-awareness unit	Introduction to emotions	Focused breathing exercises	Quarter-note rhythm Eighth-note rhythm Sixteenth-note rhythm
	Emotional awareness	Focused breathing exercises	Quarter-note rhythm Eighth-note rhythm Sixteenth-note rhythm
	Emotion regulation	Focused breathing exercises	Quarter-note rhythm Eighth-note rhythm Sixteenth-note rhythm
Social awareness unit	Perspective-taking	Active listening activities	Quarter-note rhythm Eighth-note rhythm Sixteenth-note rhythm
	Empathy toward others	Mindfulness reflective	Quarter-note rhythm Eighth-note rhythm Sixteenth-note rhythm
	Anger management	Mindfulness meditation	Quarter-note rhythm Eighth-note rhythm Sixteenth-note rhythm
	Conflict management	Mindfulness reflective	Quarter-note rhythm Eighth-note rhythm Sixteenth-note rhythm
	Conflict resolution	Mindfulness reflective	Quarter-note rhythm Eighth-note rhythm Sixteenth-note rhythm
Responsible decision-making unit	Positive life choices	Mindful movement	Quarter-note rhythm Eighth-note rhythm Sixteenth-note rhythm
	Ethical choices based on awareness and compassion	Community service learning	Quarter-note rhythm Eighth-note rhythm Sixteenth-note rhythm

## 4 Study experiment

### 4.1 Participants

This study recruited 294 male students from a fire service university in China (Although recruitment was not gender-specific, the high proportion of male students in the firefighting school resulted in all participants being male). Participants ranged in age from 18 to 25 years and were screened through internal psychological assessments at the school to identify those with mild to moderate anxiety, depression, and stress imbalances. Initially, participants were grouped into three major categories based on their emotional types: depression, anxiety, and stress imbalance, with 105, 99, and 90 individuals in each group, respectively. Subsequently, each of these major groups was subdivided into three different subgroups based on rhythmic music types: quarter-note rhythm, eighth-note rhythm, and sixteenth-note rhythm. Participants within the major groups were randomly and evenly assigned to the three different subgroups, resulting in subgroup sizes of 35, 33, and 30 participants, respectively. The allocation of participant groups is shown in Table 2.

**Table 2:** Participant group assignments.

**Participant group assignments**									
**Major emotional types**	**Depression group**			**Anxiety group**			**Stress imbalance group**		
**Number of participants in major groups (** ***N*** **)**	**105**			**99**			**90**		
Subgroups of emotional types	A	B	C	A	B	C	A	B	C
Number of participants in subgroups (*N*)	35	35	35	33	33	33	30	30	30

### 4.2 Data measurement methods

This section introduces the statistical methods and emotional measurement scales used in the case study experiment. To assess the three primary emotional health issues affecting university students, this study utilized the standardized Beck Anxiety Inventory (BAI), the Center for Epidemiologic Studies Depression Scale (CES-D), and the Perceived Stress Scale (PSS) to collect emotional health data from participants before and after the course. Additionally, academic performance data were gathered through professional testing conducted before and after the experiment. The collected data were analyzed using two-way ANOVA with the statistical software SPSS. Additionally, the study conducted a text coding analysis of the self-reflection reports submitted by students after completing the three chapters of the course.

#### 4.2.1 Two-way analysis of variance

Two-way Analysis of Variance (ANOVA) is commonly used to determine whether different levels of two factors have a significant effect on the outcome, as well as to explore potential interactions between factors in various experimental designs. The use of two-way ANOVA allows researchers to examine the main effects of each factor and whether these factors interact in influencing the dependent variable. Therefore, two-way ANOVA is a crucial tool for investigating the combined impact of two categorical independent variables on a continuous outcome (Kim, 2014).

#### 4.2.2 Beck Anxiety Inventory

The Beck Anxiety Inventory (BAI) was developed by Aaron Beck in 1985 and its validity has been confirmed in multiple studies. In this study, it is used to measure anxiety levels. The BAI consists of 21 questions covering cognitive, emotional, and physical dimensions. Responses are given on a 4-point Likert scale, with higher scores indicating more severe anxiety symptoms. Anxiety intensity is categorized as follows: minimal anxiety (0–7 points), mild anxiety (8–15 points), moderate anxiety (16–25 points), and severe anxiety (26–63 points). Regarding reliability, the BAI has a Cronbach's alpha of 0.90 (Kwon and Oei, 1992).

#### 4.2.3 Center for Epidemiologic Studies Depression Scale

The study utilized the Center for Epidemiological Studies Depression Scale (CES-D) to measure depression (Radloff, 1977). The CES-D was introduced in China in the 1990s, and its validity has been tested in multiple studies (Liu et al., 2020). The CES-D is a self-reported depression scale developed by Radloff (1977) to assess depressive symptoms in the general population. To reduce interview time and the burden of answering questions, Kohout et al. (1993) developed a short version of the CES-D based on the results of exploratory factor analysis, selecting items with high factor loadings across various factors. The short version contains nine items, rated on a 4-point Likert scale, with higher scores indicating greater levels of depression. The critical scores used to identify clinical depression are as follows: normal (0–15 points), possible depression (16–24 points), and major depression (≥25 points) (Park and Kim, 2011). Regarding reliability, the CES-D has a Cronbach's alpha of 0.90 (Cho, 1993).

#### 4.2.4 Perceived Stress Scale

The Perceived Stress Scale (PSS) is one of the most widely used tools for assessing perceived stress globally (Park et al., 2014). Initially developed by Cohen et al. in 1983 (Yoo, 2005), the scale aims to measure the extent of stress experienced by individuals in unpredictable, uncontrollable, and overloaded situations. The original version, PSS-14, contains 14 items, with seven negatively phrased questions (e.g., “How often have you felt unable to control important things in your life?”) and seven positively phrased questions (e.g., “How confident have you felt in your ability to handle personal issues?”). Chung et al. (2023) confirmed that the Chinese version of the Perceived Stress Scale (CPSS-14) has high reliability. The scale includes 14 items rated on a 4-point Likert scale, with higher scores indicating higher perceived stress levels. Stress intensity is categorized as follows: normal stress range (0–28 points), elevated stress (29–42 points), and severe stress (43–56 points). In this study, CPSS-14 was used for experimental measurement.

#### 4.2.5 Firefighting assessment test

The firefighting test questions were collaboratively developed by three instructors with 6–10 years of experience. The questions primarily focus on five areas: fundamental firefighting theory, operational knowledge, safety in training, emergency response, and overall competency. The assessment consists of two sets of exam papers, designated as Paper A and Paper B, with uniform question types including single-choice, multiple-choice, and true/false questions, each containing ten items, for a total score of 100 points. To ensure consistency in the baseline between the two testing occasions, both exam papers are of moderate difficulty, closely aligned in content, and balanced in the topics assessed. The single-choice questions combine theoretical knowledge with practical application, focusing on the foundational qualities of firefighting students. The multiple-choice questions integrate scenario simulations with emergency response, emphasizing the comprehensive abilities of firefighting students. The true/false questions are designed around safety regulations in three firefighting scenarios: training, duty, and combat, focusing on students' ability to identify and assess risks. Overall, the test comprehensively covers various aspects of the firefighting curriculum and provides a scientifically objective representation of the learning outcomes for firefighting students.

#### 4.2.6 Self-reflection report coding

The self-reflection reports are designed to clarify the patterns of emotional transitions and timing for students experiencing emotional issues such as depression, anxiety, and stress imbalances during their participation in the SEL program, aiding teachers in managing the emotional transitions of different student groups. The study primarily presents the written texts, with emotional coding conducted through the extraction of key emotional vocabulary, qualitative adjectives, and degree adverbs. Since our research aims to explore the patterns of emotional transitions related to depression, anxiety, and stress imbalances, we extracted emotional vocabulary that describes these symptoms from relevant journal articles, psychological reports, and previous patient self-reports. Examples of such emotional vocabulary include terms like “nervous,” “despair,” and “helplessness.” Additionally, we incorporated a highly representative list of words for each emotional type, summarized by Lin and Yao (2016), to better categorize the emotional keywords by their respective emotional types. To quantify emotional changes, the study also collected a range of qualitative adjectives and degree adverbs that describe variations in intensity, such as “rarely,” “a little,” “fairly,” “very,” “good,” and “poor.” This vocabulary was combined with the emotional words to comprehensively determine the timing of emotional changes. The degree of emotional transition was encoded based on emotional vocabulary and degree adverbs, alongside contextual analysis. Time was categorized based on course chapters, designated as the early, middle, and late sections, corresponding to the first, second, and third chapters, respectively. Researchers calculated the frequency of emotional transition vocabulary mentioned by participants in different subgroups within each chapter, and combined this with the timing to establish the encoding position on a coordinate axis. The specific coding system is shown in Table 3.

**Table 3:** Self-reflection report coding system.

**Timeline**			**Emotional keywords**			**Qualitative adjectives**			**Degree adverbs**	
**Early-stage (chapter one)**	**Mid-stage (chapter two)**	**Later-stage (chapter three)**	**Depression**	**Anxiety**	**Stress imbalance**	**Low degree change adverbs**	**Medium degree change adverbs**	**High degree change adverbs**	**Positive adjectives**	**Negative adjectives**
Introduction to emotions Emotional awareness Emotion regulation	Perspective-taking Empathy toward others Anger management Conflict management Conflict resolution	Positive life choices Ethical choices based on awareness and compassion	Sadness; frustration; helplessness; unhappiness; despair; loneliness; unhappiness; depression; uselessness; undermined self-esteem: shame; worthlessness	Nervousness; chest tightness; shortness of breath; dizziness; vertigo; fear; fainting; chest pain; heartbeat; increased respiratory rate; distracted or unsettled mind	Tension; oppression; fatigue; despair; irritability; agitation	A little bit; a bit; some; a few; somewhat; slightly; slightly; more	Even more; very; increasingly; especially; particularly	Most; extremely; too; very; almost; nearly	Good; well; beneficial; fine; superior; improvement; turnaround; positive change	Poor; deterioration; severe; serious

### 4.3 Implementation of the mindfulness-integrated music rhythm program

Participants were instructed to follow these steps during the mindfulness breathing exercises: (1) adopt any comfortable position—sitting, lying down, or standing—while maintaining focus on their breathing; (2) fully experience the physical sensations and emotional changes brought by each breath; (3) keep their attention on the breath, and if they notice their mind wandering, take a deep breath to refocus on their breathing. Within each major group categorized by type of emotional issue, participants were further randomly assigned to one of three subgroups practicing mindfulness with different low-frequency music rhythms: quarter-note, eighth-note, or sixteenth-note rhythms. During mindfulness sessions, participants were instructed to use an audio playback device (e.g., smartphone, computer, or speaker) to play the assigned rhythm before starting the mindfulness exercise to enhance their practice.

### 4.4 Procedure and design

Recruitment advertisements were posted across the university campus, inviting students interested in participating in the mindfulness-integrated music rhythm SEL program to provide their personal information. A total of 294 participants were then grouped according to their emotional issues: depression (105 participants), anxiety (99 participants), and stress imbalance (90 participants). Subsequently, within each major group, participants were randomly assigned to three experimental subgroups, with 35, 33, and 30 participants per subgroup, respectively. The experiment was jointly conducted by teachers and researchers who held a National Level 2 Psychological Counselor Certificate and had 5 years of mindfulness training experience, along with previous involvement in SEL courses. Before the semester began, we provided teachers, school personnel, and community workers with a SEL teaching syllabus and instructional plan that included the program themes. Digital copies of the specific SEL curriculum plan and music samples were sent in advance to the school coordinators, community workers, and teachers. Teachers then presented the curriculum plan and music samples to the students and coordinated with them to support the existing SEL programs and activities.

**Figure 1:**
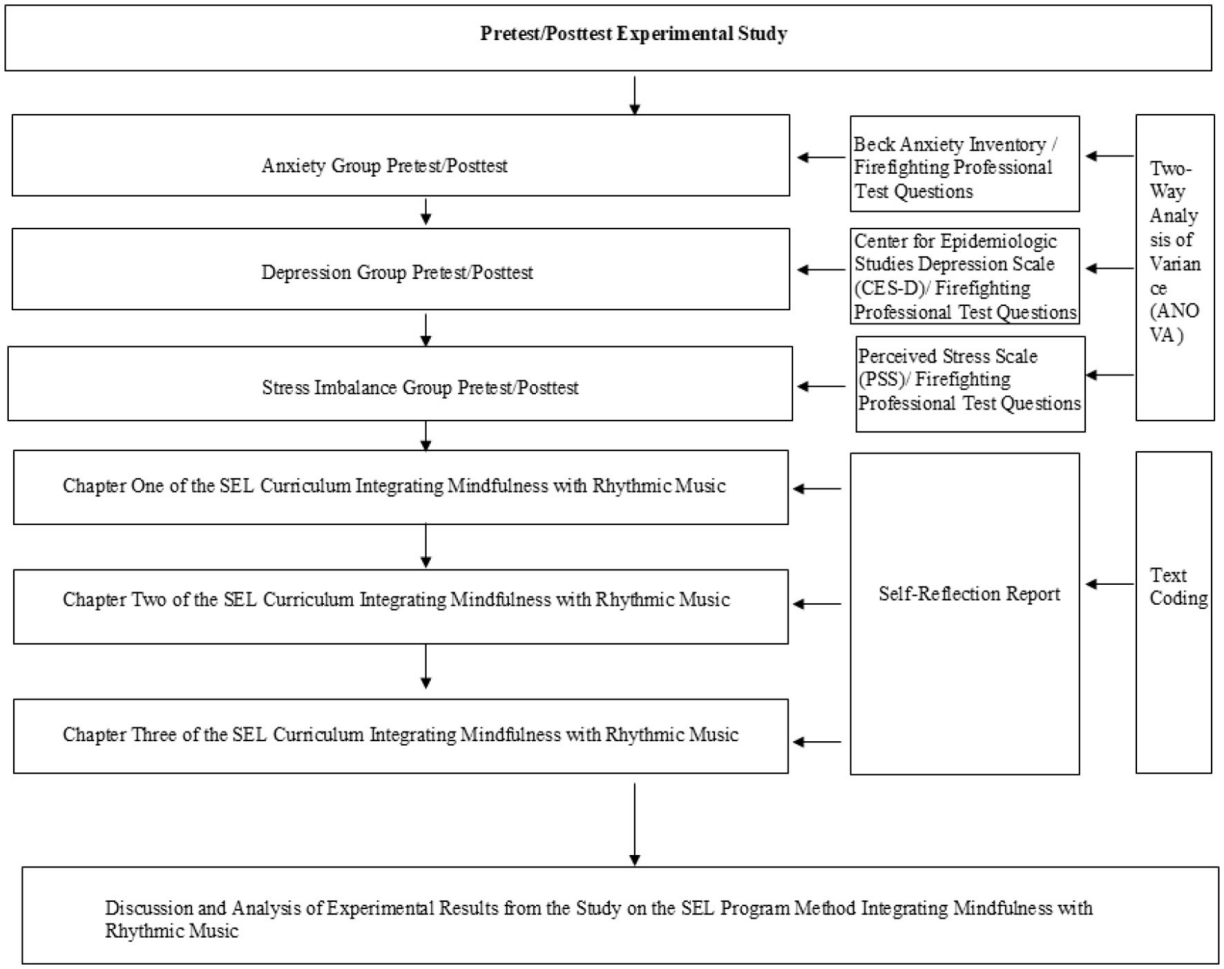
Research flowchart.

The study employed a pre-test/post-test experimental design to explore whether different musical rhythms have differential effects on the improvement of students with depression, anxiety, and stress imbalances, and whether the alleviation of emotional issues in students would promote improvements in their academic performance. Additionally, the study incorporated students' self-reflection reports as a method for exploring the patterns of emotional transitions among students. The specific research process is illustrated in Figure 1.

Pre-Test/Post-Test Experiment: Since this course was conducted online, participants were instructed to select a quiet, undisturbed room for the experiment. At the beginning of the experiment, each participant was informed that they were taking part in a study related to social-emotional skills. Researchers provided detailed instructions, ensuring that participants fully understood the experimental procedures before proceeding. During the pre-test phase, participants completed the following questionnaires and assessments: (1) Beck Anxiety Inventory (BAI); (2) Center for Epidemiologic Studies Depression Scale (CES-D); (3) Perceived Stress Scale (PSS); (4) Firefighting Professional Test (Form A). Each questionnaire required ~20 min to complete, while the professional test took about 30 min. Participants then underwent a semester-long SEL program. At the end of the course, posttest assessments were conducted, during which participants completed the same questionnaires and the Firefighting Professional Test (Form B). The study employed two-way ANOVA, simple effects analysis, means, and standard deviations to analyze the questionnaire data. Data analysis was conducted using SPSS, with a significance threshold set at *p* < 0.05.

Self-Reflection Report Analysis: At the start of the experiment, each participant was required to complete a written reflection report after the conclusion of each chapter, with a word limit of 100 words. The reflective reports included issues related to emotional alleviation, social skills/behavior growth, and learning outcomes. These data were used to address the research questions: (1) How did participants' emotions gradually change after participating in the SEL program? (2) What impact did this have on participants' learning outcomes? (3) Did students subjectively feel that their emotional issues were alleviated? The coding of the self-reflection reports was conducted manually, with participation from both researchers and teachers. Consistency among coders was achieved through consensus verification. All coders reviewed and coded each text according to the coding framework outlined in Table 3. In cases of disagreement among coders, discussions were held until a consensus was reached between the two primary coders regarding the selected labels. If disagreements could not be resolved within the primary coding team, an external third coder (the third author of this paper) was consulted to make the final decision. Throughout this process, no instances arose where consensus could not be achieved. This study received ethical approval from the ethics committee of Huaqiao University, with the research protocol undergoing careful review. Written informed consent was obtained from participants, and measures were taken to ensure the confidentiality of research data in accordance with the ethical guidelines of the Chinese Psychological Society.

### 4.5 Experimental music samples production

**Figure 2:**
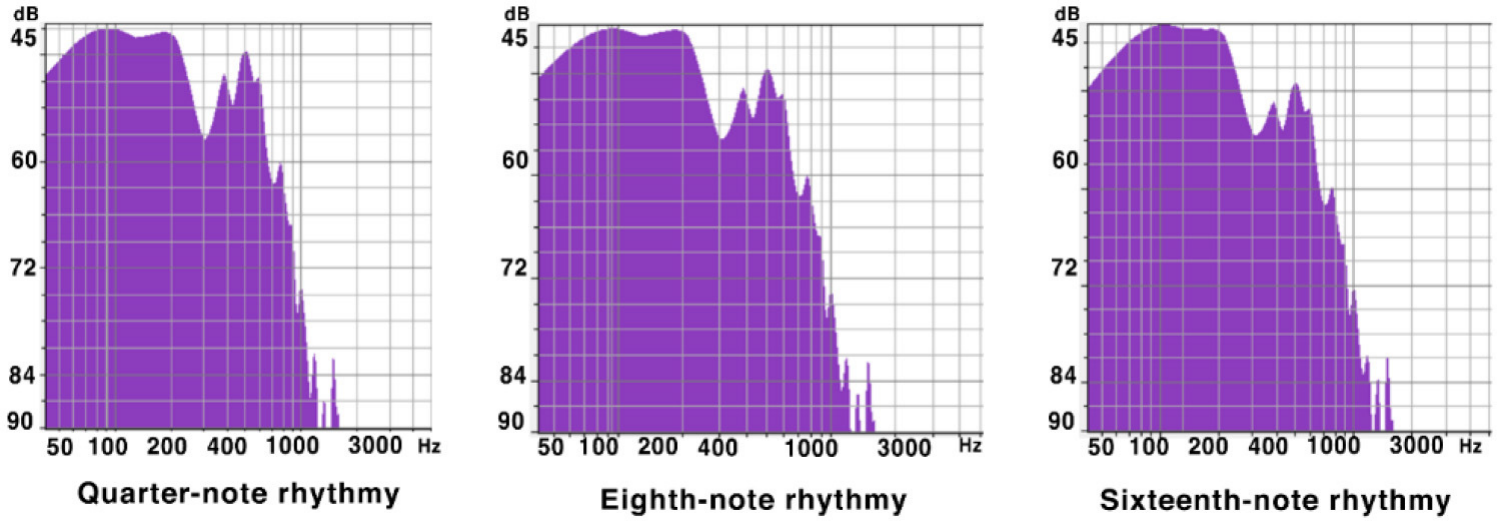
Music rhythm spectrogram.

**Figure 3:**
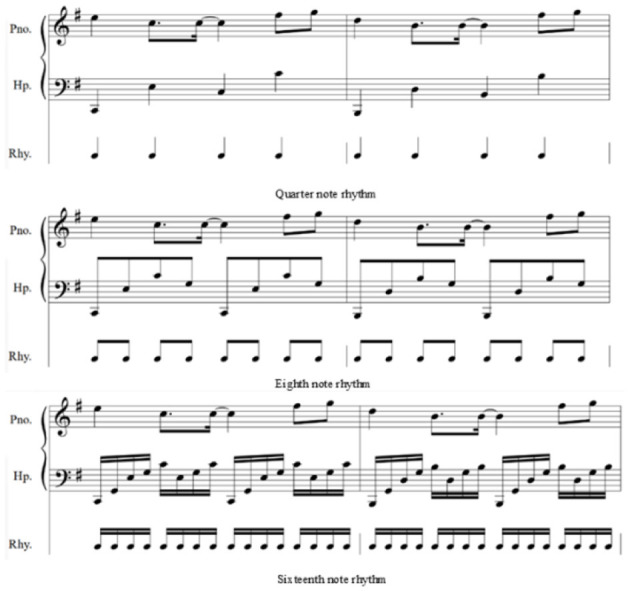
Musical score example.

The experimental samples were re-arranged by a composition teacher with 6 years of teaching experience, focusing on the predominant rhythms of quarter notes, eighth notes, and sixteenth notes. The selection of these three musical rhythms is based on two reasons. First, musical rhythms can be divided into predominant and subordinate rhythms, which function similarly to stable and unstable tones in a scale. The quarter, eighth, and sixteenth note rhythms serve as the predominant musical rhythms. A quarter note can be divided into two eighth notes, and an eighth note can be subdivided into two sixteenth notes, thereby forming smaller rhythmic units. Second, research indicates that music with eighth to sixteenth note rhythms can effectively alleviate symptoms of depression, while relaxed rhythms are beneficial for easing anxiety and stress. Quarter note rhythms are generally slower than eighth note rhythms (Kwon et al., 2017; Elliott et al., 2011; Bokiev et al., 2018; Joshi and Kiran, 2020; Feng, 2022). Additionally, the samples were set to a duration of 5 min, as this is a common length for modern songs. Soft and quiet sounds are considered particularly relaxing. Among musical instruments, the piano and string instruments are regarded as especially soothing. Therefore, the experimental samples were arranged with the harp as the accompanying instrument and the piano as the melodic instrument. The harp, as a plucked instrument, generates vibrations in the lower register through the strings interacting with the air, providing a large dynamic range and a more resonant sound than the piano. The piano, with its superior timbre in the mid to high registers, complements the harp well. Moreover, it has been found that low-frequency music (defined as music typically below 380 Hz) can promote psychological balance and assist individuals in achieving relaxation or meditative states; thus, the study selected suitable low-frequency music as the experimental stimulus samples. Additionally, other fundamental musical elements such as harmony, dynamics, tempo, texture, and timbre remained unchanged in the experimental samples apart from rhythm. The composition teacher initially selected a lower range for the mid and bass registers: G1-A5. In the later stages of music editing, an equalizer (EQ) was employed to enhance the low frequencies and reduce the mid to high-frequency signals, thereby highlighting the low-frequency aspects of the music. Figure 2 presents the frequency analysis of the final experimental samples for the quarter note, eighth note, and sixteenth note rhythms. Examples of the sheet music are shown in Figure 3, and the arrangement information is detailed in Table 4.

**Table 4:** Music rhythm design details.

**Rhythm**	**Time**	**Tune**	**Melody instruments**	**Accompaniment of musical instruments**
Quarter note rhythm	5 min	G Major	Piano	Harp
Eighth note rhythm	5 min	G Major	Piano	Harp
Sixteenth note rhythm	5 min	G Major	Piano	Harp

## 5 Results

### 5.1 Pre- and post-experiment data for the anxiety group

#### 5.1.1 Anxiety levels of the anxiety group before and after the experiment

The anxiety group utilized a two-way analysis of variance (ANOVA) to examine the relationship between the effects of the three subgroups on anxiety levels before and after the experiment, as presented in Table 5 (where A, B, and C represent the quarter-note rhythm subgroup, eighth-note rhythm subgroup, and sixteenth-note rhythm subgroup, respectively). The experiment showed significance (*F* = 85.661, *p* = 0.000 <0.05), indicating that the experiment had a differential effect on anxiety, and there were differences in anxiety levels among the three subgroups. Additionally, the interaction between the experiment and the three groups was significant (*F* = 6.247, *p* = 0.002 <0.05), prompting further analysis of the second-order effects as shown in the subsequent effect table. The post-experiment anxiety level scores for the three subgroups were 13.45 ± 3.55, 18.42 ± 4.19, and 24.24 ± 6.48, respectively, indicating a reduction in anxiety levels across all groups. The pre-experiment results showed no significant differences among the quarter-note, eighth-note, and sixteenth-note rhythm subgroups. Post-experiment, all three subgroups exhibited a degree of anxiety reduction. Notably, the quarter-note rhythm subgroup demonstrated the most significant decrease in anxiety levels, followed by the eighth-note rhythm subgroup, while the sixteenth-note rhythm subgroup showed the least reduction. Moreover, the anxiety scores of the quarter-note rhythm subgroup were significantly lower than those of both the eighth-note and sixteenth-note rhythm subgroups. The eighth-note rhythm subgroup's anxiety scores were also significantly lower than those of the sixteenth-note rhythm subgroup. This indicates that the effect of stimulation in the quarter-note rhythm subgroup was stronger than that in the eighth-note rhythm subgroup, and the eighth-note rhythm subgroup was more effective than the sixteenth-note rhythm subgroup.

**Table 5:** Statistical data related to the anxiety group^**^.

**Results of two-factor analysis of variance on anxiety**						
**Source of variation**	**Sum of squares**	***df***	**Mean square**	***F***	***p***	**Partial eta squared (partial** η**2)**
Intercept	115,010.020	1	115,010.020	1,710.170	<0.001^**^	0.899
Treatment	5,760.727	1	5,760.727	85.661	<0.001^**^	0.309
Three groups	1,098.859	2	549.429	8.170	<0.001^**^	0.078
Treatment three groups	840.273	2	420.136	6.247	0.002^*^	0.061
Residual	12,912.121	192	67.251			
*R*^2^: 0.374						
**Comparison of the anxiety status of the three subgroups before and after the experiment (mean** ±**standard deviation)**						
	**A (*****n*** = **33)**		**B (*****n*** = **33)**		**C (*****n*** = **33)**	
Before treatment	29.33 ± 10.89		29.12 ± 10.46		30.03 ± 10.17	
After treatment	13.45 ± 3.55		18.42 ± 4.19		24.24 ± 6.48	
**Simple effects (before and after the experiment & three subgroups of anxiety group)**						
**Treatment**	**Three groups**	**MD**	**SE**	***t*** **-value**	***p*** **-value**	**Cohen's** ***d***
Before treatment	A–B	0.212	2.019	0.105	1.000	0.026
Before treatment	A–C	−0.697	2.019	−0.345	1.000	−0.085
Before treatment	B–C	−0.909	2.019	−0.450	1.000	−0.111
After treatment	A–B	−4.970	2.019	−2.462	0.044^*^	−0.606
After treatment	A–C	−10.788	2.019	−5.344	<0.001^**^	−1.315
After treatment	B–C	−5.818	2.019	−2.882	0.013	−0.709
**Three groups**	**Treatment**	**MD**	**SE**	***t*** **-value**	***p*** **-value**	**Cohen's** ***d***
A	Before treatment–after treatment	15.879	2.019	7.865	<0.001^**^	1.936
B	Before treatment–after treatment	10.697	2.019	5.299	<0.001^**^	1.304
C	Before treatment–after treatment	5.788	2.019	2.867	0.005^**^	0.706

^*^p < 0.05; ^**^p < 0.01.

#### 5.1.2 Academic performance of the anxiety group before and after the experiment

The impact of the experiment on the academic performance of participants in the three subgroups of the anxiety group before and after the intervention is shown in Table 6. The experiment demonstrated significance (*F* = 73.007, *p* = 0.000 <0.05), indicating significant differences in academic performance within the anxiety group. The three subgroups also showed significance (*F* = 9.075, *p* = 0.000 <0.05), indicating that there were differences in academic performance among the three subgroups. The interaction between the experiment and the three groups was significant (*F* = 10.163, *p* = 0.000 <0.05), allowing for further analysis of second-order effects. The results indicated that there were no differences in academic performance among the three groups prior to the experiment. After the intervention, all three groups showed improvements in their scores. The quarter-note rhythm subgroup achieved the highest scores, followed by the eighth-note rhythm subgroup, while the sixteenth-note rhythm subgroup had the lowest performance. Furthermore, the scores of the quarter-note rhythm subgroup were significantly higher than those of the eighth-note rhythm subgroup, and the eighth-note rhythm subgroup's scores were significantly higher than those of the sixteenth-note rhythm subgroup.

**Table 6:** Statistical data related to academic performance for the anxiety group.

**Results of two-factor analysis of variance on academic performance of anxiety group**						
**Source of variation**	**Sum of squares**	***df***	**Mean square**	***F***	***p***	**Partial eta squared (partial** η**2)**
Intercept	1,293,737.500	1	1,293,737.500	86,491.240	<0.001^**^	0.998
Treatment	1,092.045	1	1,092.045	73.007	<0.001^**^	0.275
Three groups	271.485	2	135.742	9.075	<0.001^**^	0.086
Treatment three groups	304.030	2	152.015	10.163	<0.001^**^	0.096
Residual	2,871.939	192	14.958			
*R*^2^: 0.374						
**Comparison of the mean scores of the three subgroups of the anxiety group before and after the experiment (mean** ±**standard deviation)**						
	**A (*****n*** = **33)**		**B (*****n*** = **33)**		**C (*****n*** = **33)**	
Before treatment	78.12 ± 3.94		79.06 ± 3.70		78.27 ± 3.46	
After treatment	86.12 ± 4.23		83.12 ± 4.23		80.30 ± 3.57	
**Simple effects (before and after the experiment & three subgroups of anxiety group)**						
**Treatment**	**Three groups**	**MD**	**SE**	***t*** **-value**	***p*** **-value**	**Cohen's** ***d***
Before treatment	A–B	−0.939	0.952	−0.987	0.975	−0.243
Before treatment	A–C	−0.152	0.952	−0.159	1.000	−0.039
Before treatment	B–C	0.788	0.952	0.827	1.000	0.204
After treatment	A–B	3.000	0.952	3.151	0.006^**^	0.776
After treatment	A–C	5.818	0.952	6.111	<0.001^**^	1.504
After treatment	B–C	2.818	0.952	2.960	0.010	0.729
**Three groups**	**Treatment**	**MD**	**SE**	***t*** **-value**	***p*** **-value**	**Cohen's** ***d***
A	Before treatment–after treatment	−8.000	0.952	−8.402	<0.001^**^	−2.068
B	Before treatment–after treatment	−4.061	0.952	−4.265	<0.001^**^	−1.050
C	Before treatment–after treatment	−2.030	0.952	−2.132	0.034^*^	−0.525

^*^p < 0.05; ^**^p < 0.01.

### 5.2 Pre- and post-experiment data for the depression group

#### 5.2.1 Depression levels of the depression group before and after the experiment

The analysis of the depression group revealed significant differences in pre- and post-experiment depression levels (*F* = 78.511, *p* = 0.000 <0.05), indicating that the intervention had a significant impact on depression levels, as shown in Table 7. Additionally, a significant interaction effect was found between the intervention and the three subgroups (*F* = 3.855, *p* = 0.023 <0.05), prompting further second-order effect analysis. Table 7 shows that there were no significant differences in depression levels among the three subgroups before the intervention. After the intervention, depression levels in all three subgroups improved compared to pre-intervention levels. Notably, the eighth-note rhythm subgroup experienced the greatest reduction in depression levels. Since the depression scores for the quarter-note subgroup were higher than those of the sixteenth-note subgroup, the sixteenth-note subgroup showed greater reduction compared to the quarter-note subgroup. These results indicate that the quarter-note subgroup experienced the least improvement in depression relief among the three subgroups.

**Table 7:** Statistical data related to the depression group^**^.

**Results of two-factor analysis of variance on anxiety**						
**Source of variation**	**Sum of squares**	***df***	**Mean square**	***F***	***p***	**Partial eta squared (partial** η**2)**
Intercept	111,136.005	1	111,136.005	8,053.170	<0.001	0.975
Treatment	1,083.471	1	1,083.471	78.511	<0.001	0.278
Three groups	65.867	2	32.933	2.386	0.095	0.023
Treatment three groups	106.400	2	53.200	3.855	0.023^*^	0.036
Residual	2,815.257	204	13.800			
*R*^2^: 0.308						
**Comparison of the means of depression in the three subgroups before and after the experiment in the depression group(mean** ±**standard deviation)**						
	**A (*****n*** = **35)**		**B (*****n*** = **35)**		**C (*****n*** = **35)**	
Before treatment	20.49 ± 3.75		20.94 ± 4.30		20.77 ± 4.00	
After treatment	26.23 ± 2.85		23.49 ± 3.79		26.11 ± 3.42	
**Simple effects (before and after the experiment & three subgroups of anxiety group)**						
**Treatment**	**Three groups**	**MD**	**SE**	***t*** **-value**	***p*** **-value**	**Cohen's** ***d***
Before treatment	A–B	−0.457	0.888	−0.515	1.000	−0.123
Before treatment	A–C	−0.286	0.888	−0.322	1.000	−0.077
Before treatment	B–C	0.171	0.888	0.193	1.000	0.046
After treatment	A–B	2.743	0.888	3.089	0.007	0.738
After treatment	A–C	0.114	0.888	0.129	1.000	0.031
After treatment	B–C	−2.629	0.888	−2.960	0.010	−0.708
**Three groups**	**Treatment**	**MD**	**SE**	***t*** **-value**	***p*** **-value**	**Cohen's** ***d***
A	Before treatment–after treatment	−5.743	0.888	−6.467	<0.001	−1.546
B	Before treatment–after treatment	−2.543	0.888	−2.863	0.005	−0.685
C	Before treatment–after treatment	−5.343	0.888	−6.017	<0.001	−1.438

^*^p < 0.05; ^**^p < 0.01.

#### 5.2.2 Academic performance of the depression group before and after the experiment

The research results are presented in Table 8, which shows the impact of the intervention on academic performance among the three subgroups within the depression group. The findings indicate significant differences in scores before and after the experiment (*F* = 41.314, *p* = 0.000 <0.05), suggesting that changes occurred in academic performance between the pre-experiment and post-experiment assessments. However, there were no significant differences among the three subgroups (*F* = 2.173, *p* = 0.116 > 0.05), indicating that the academic performance of the three subgroups did not exhibit any differential effects.

**Table 8:** Statistical data related to academic performance for the depression group.

**Results of two-factor analysis of variance on academic performance of depression group**						
**Source of variation**	**Sum of squares**	***df***	**Mean square**	***F***	***p***	**Partial eta squared (partial** η**2)**
Intercept	1,327,093.505	1	1,327,093.505	65,270.490	<0.001^**^	0.997
Treatment	840.000	1	840.000	41.314	<0.001^**^	0.168
Three groups	88.352	2	44.176	2.173	0.116	0.021
Treatment three groups	18.371	2	9.186	0.452	0.637	0.004
Residual	4,147.771	204	20.332			
*R*^2^:0.186						
**Comparison of the mean scores of the three subgroups of the depression group before and after the experiment (mean** ±**standard deviation)**						
	**A (*****n*** = **35)**		**B (*****n*** = **35)**		**C (*****n*** = **35)**	
Before treatment	77.03 ± 4.39		77.46 ± 5.08		78.00 ± 4.30	
After treatment	80.20 ± 4.53		81.97 ± 4.36		82.31 ± 4.34	

^**^p < 0.01.

### 5.3 Pressure group experimental data results

#### 5.3.1 Pressure levels before and after the experiment

The relationships between the impact of the quarter-note rhythm subgroup, eighth-note rhythm subgroup, and sixteenth-note rhythm subgroup on pressure levels before and after the experiment are shown in Table 9. The results indicate significant differences before and after the experiment (*F* = 63.725, *p* = 0.000 <0.05), suggesting that the intervention had a differential effect on pressure levels. Prior to the experiment, there were no significant differences among the three subgroups (*F* = 2.768, *p* = 0.066 > 0.05), indicating no differences in pressure levels before the intervention. Post-experiment, there was a significant difference in pressure levels, and the interaction between time and group was also significant (*F* = 3.514, *p* = 0.032 <0.05), allowing for further analysis of second-order effects. As shown in Table 9, there were no differences in pressure levels among the three subgroups before the experiment. After the intervention, all three subgroups exhibited reductions in pressure levels. Notably, there were no differences between the quarter-note and eighth-note rhythm subgroups, while the pressure level in the sixteenth-note rhythm subgroup was significantly lower than that in both the quarter-note and eighth-note rhythm subgroups. This indicates that the reduction in pressure levels in the sixteenth-note subgroup was more effective than in the quarter-note and eighth-note subgroups.

**Table 9:** Statistical data related to the pressure group^**^.

**Two-factor variance analysis results of stress conditions**						
**Source of variation**	**Sum of squares**	***df***	**Mean square**	***F***	***p***	**Partial eta squared (partial** η**2)**
Intercept	151,728.200	1	151,728.200	2,158.237	<0.001	0.925
Treatment	4,480.022	1	4,480.022	63.725	<0.001	0.268
Three groups	389.200	2	194.600	2.768	0.066	0.031
Treatment three groups	494.044	2	247.022	3.514	0.032^*^	0.039
Residual	12,232.533	174	70.302			
*R*^2^: 0.305						
**Comparison of the mean values of the stress conditions of the three subgroups before and after the experiment in the stress group (mean** ±**standard deviation)**						
	**A (*****n*** = **30)**		**B (*****n*** = **30)**		**C (*****n*** = **30)**	
Before treatment	33.77 ± 10.72		34.03 ± 11.80		34.27 ± 9.71	
After treatment	26.77 ± 4.55		25.70 ± 4.65		19.67 ± 5.56	
**Simple effects (before and after the experiment & three subgroups of anxiety group)**						
**Treatment**	**Three groups**	**MD**	**SE**	***t*** **-value**	***p*** **-value**	**Cohen's** ***d***
Before treatment	A–B	−0.267	2.165	−0.123	1.000	−0.032
Before treatment	A–C	−0.500	2.165	−0.231	1.000	−0.060
Before treatment	B–C	−0.233	2.165	−0.108	1.000	−0.028
After treatment	A–B	1.067	2.165	0.493	1.000	0.127
After treatment	A–C	7.100	2.165	3.280	0.004	0.847
After treatment	B–C	6.033	2.165	2.787	0.018	0.720
**Three groups**	**Treatment**	**MD**	**SE**	***t*** **-value**	***p*** **-value**	**Cohen's** ***d***
A	Before treatment–after treatment	7.000	2.165	3.233	0.001	0.835
B	Before treatment–after treatment	8.333	2.165	3.849	<0.001	0.994
C	Before treatment–after treatment	14.600	2.165	6.744	<0.001	1.741

^*^p < 0.05; ^**^p < 0.01.

#### 5.3.2 Academic performance results for the pressure group before and after the experiment

The relationships between the impact of the three subgroups within the pressure group on academic performance before and after the experiment are shown in Table 10. The results indicated significant differences in scores before and after the experiment (*F* = 46.811, *p* = 0.000 <0.05), suggesting that the intervention influenced academic performance. Prior to the experiment, there were no significant differences among the subgroups (*F* = 1.714, *p* = 0.183 > 0.05), indicating that there were no differences in scores among the subgroups before the intervention. Post-experiment, significant differences were observed, and the interaction between time and the three subgroups also showed significance (*F* = 3.319, *p* = 0.039 <0.05), allowing for further analysis of second-order effects. The results indicated that, compared to the pre-experiment scores, all three subgroups showed improvements after the intervention. Notably, there were no differences in scores between the quarter-note rhythm subgroup and the eighth-note rhythm subgroup, while the scores of the sixteenth-note rhythm subgroup were significantly higher than those of both the quarter-note and eighth-note rhythm subgroups.

**Table 10:** Statistical data related to academic performance for the pressure group.

**Results of two-factor analysis of variance on academic performance of anxiety group**						
**Source of variation**	**Sum of squares**	***df***	**Mean square**	***F***	***p***	**Partial eta squared (partial** η**2)**
Intercept	1,060,915.339	1	1,060,915.339	75,982.412	<0.001^**^	0.998
Treatment	653.606	1	653.606	46.811	<0.001^**^	0.212
Three groups	47.878	2	23.939	1.714	0.183	0.019
Treatment three groups	92.678	2	46.339	3.319	0.039^*^	0.037
Residual	2,429.500	174	13.963			
*R*^2^: 0.246						
**Comparison of the mean scores of the three subgroups of the anxiety group before and after the experiment (mean** ±**standard deviation)**						
	**A (*****n*** = **30)**		**B (*****n*** = **30)**		**C (*****n*** = **30)**	
Before TREATMENT	75.13 ± 3.17		74.87 ± 3.16		74.60 ± 3.22	
After treatment	77.60 ± 3.31		78.03 ± 4.16		80.40 ± 5.01	
**Simple effects (before and after the experiment & three subgroups of anxiety group)**						
**Treatment**	**Three groups**	**MD**	**SE**	***t*** **-value**	***p*** **-value**	**Cohen's** ***d***
Before treatment	A–B	0.267	0.965	0.276	1.000	0.071
Before treatment	A–C	0.533	0.965	0.553	1.000	0.143
Before treatment	B–C	0.267	0.965	0.276	1.000	0.071
After treatment	A–B	−0.433	0.965	−0.449	1.000	−0.116
After treatment	A–C	−2.800	0.965	−2.902	0.013^*^	−0.749
After treatment	B–C	−2.367	0.965	−2.453	0.045^*^	−0.633
**Three groups**	**Treatment**	**MD**	**SE**	***t*** **-value**	***p*** **-value**	**Cohen's** ***d***
A	Before treatment–after treatment	−2.467	0.965	−2.557	0.011	−0.660
B	Before treatment–after treatment	−3.167	0.965	−3.282	<0.001^**^	−0.847
C	Before treatment–after treatment	−5.800	0.965	−6.012	<0.001^**^	−1.552

^*^p < 0.05; ^**^p < 0.01.

### 5.4 Self-reflection report coding diagram

The study compiled participants' self-reflection reports and categorized them into three groups based on depression, anxiety, and stress imbalances, exploring the impact of different musical rhythms on the emotional changes among participants in the various subgroups. Furthermore, we compared the three groups of depression, anxiety, and stress imbalances to reveal the differences in emotional changes associated with different emotional issues.

#### 5.4.1 Self-reflection report coding diagram for the anxiety group

**Figure 4:**
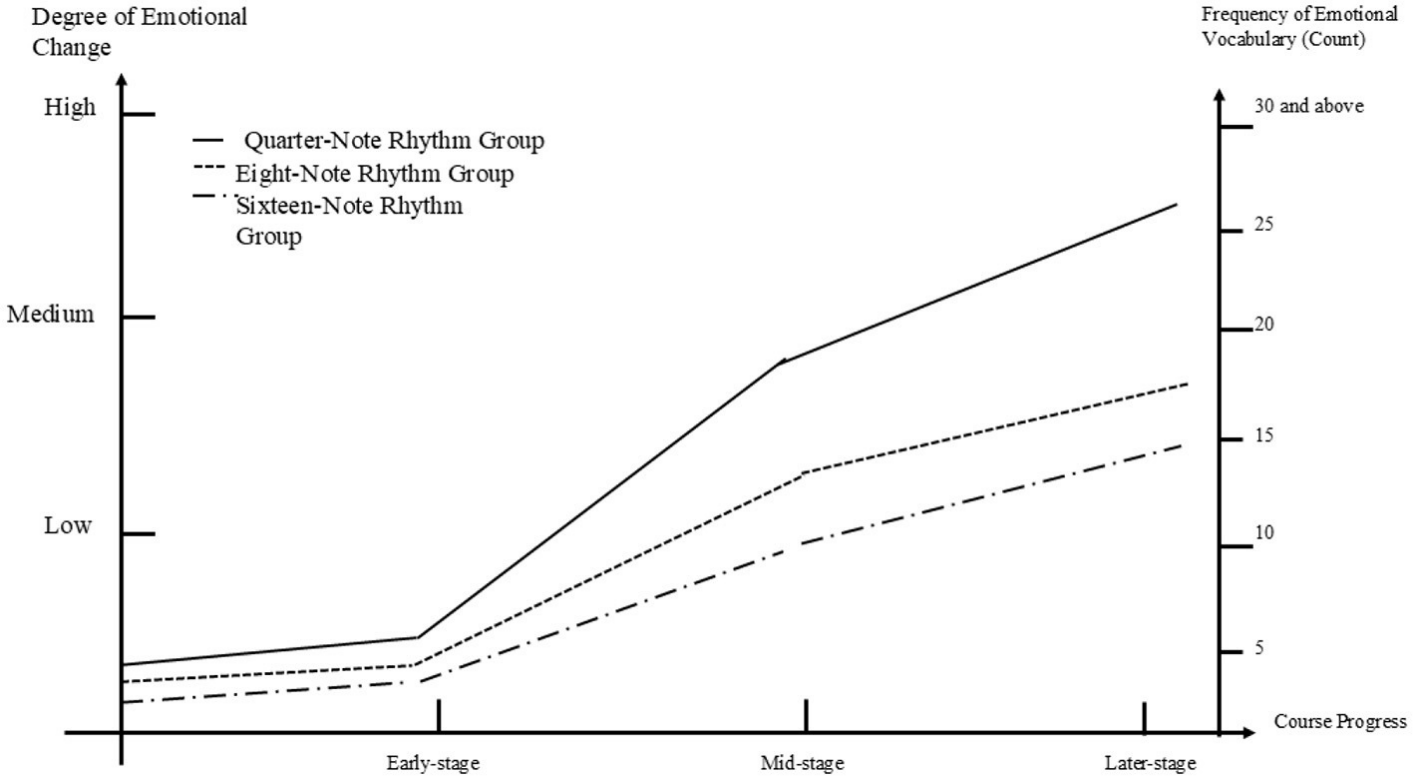
Coding for the anxiety group.

Figure 4 illustrates the process of emotional transitions among participants in the three subgroups within the anxiety group. Initially, all three subgroups exhibited a positive trend in emotional transitions. Although the three groups showed low levels of change in the early phase, it was surprising to note that by the mid-phase, all participants' emotions shifted rapidly from low to moderate levels. The trend of continued improvement was maintained in the later phase. Among the subgroups, the quarter-note rhythm subgroup displayed the most significant emotional transition compared to the eighth-note and sixteenth-note subgroups. In the early phase, the quarter-note subgroup already demonstrated better changes than the other two groups, with a frequency of seven occurrences of low-level emotional vocabulary. The mid-phase represented a turning point for the quarter-note subgroup, with a notable increase in positive emotions, reflected by 19 occurrences of moderate-level emotional vocabulary. The later phase continued this upward trend, culminating in a frequency of 27 occurrences of high-level emotional vocabulary. In contrast, the sixteenth-note subgroup exhibited the most mediocre change, with a transition from four occurrences of low-level emotional vocabulary in the early phase to 15 occurrences of moderate-level emotional vocabulary in the final phase, indicating only a shift from low to moderate levels. The eighth-note subgroup demonstrated a stable but consistently improving trend, showing a breakthrough in the mid-phase; however, the level of change was relatively gradual in the later phase, with a final frequency of 18 occurrences of moderate-level emotional vocabulary.

#### 5.4.2 Self-reflection report coding diagram for the depression group

**Figure 5:**
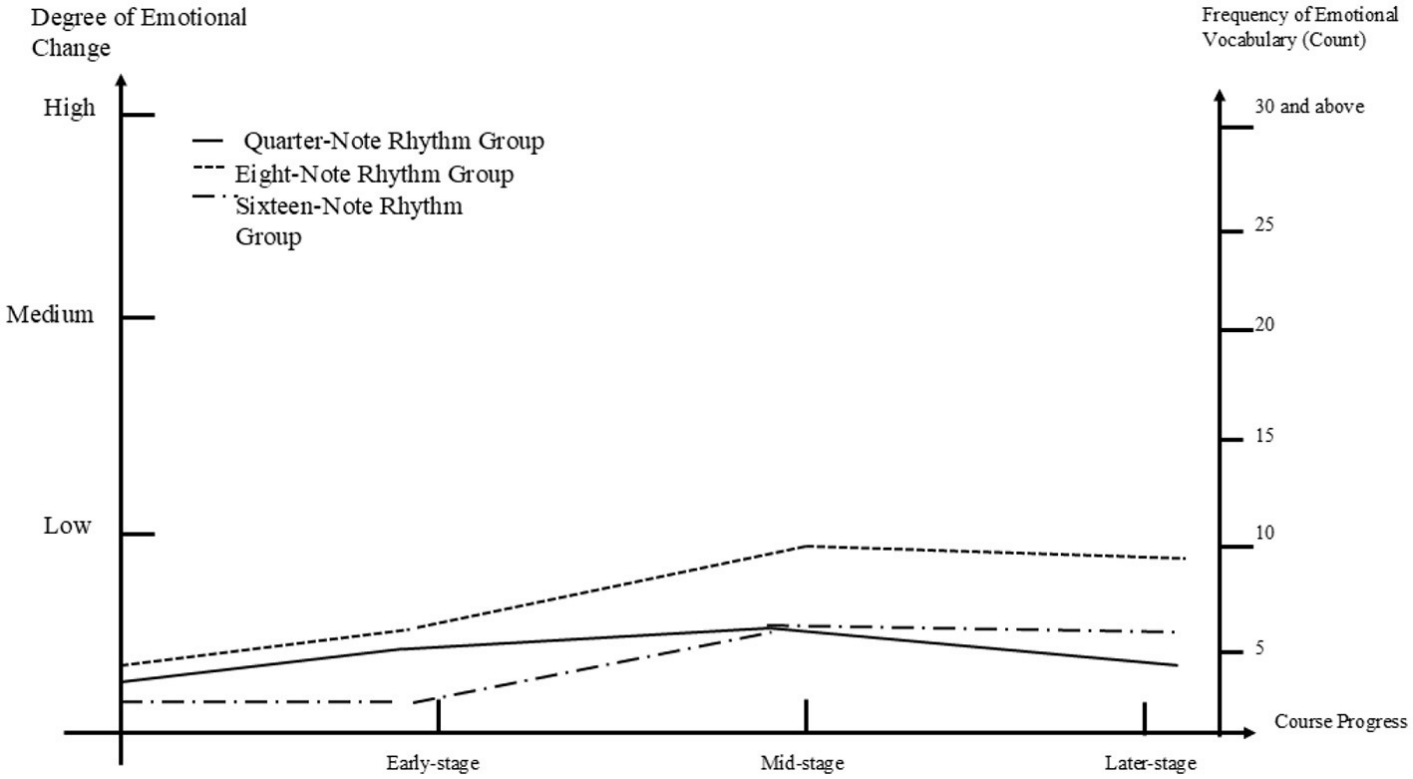
Coding for the depression group.

Figure 5 illustrates the emotional transition process of participants in the three subgroups of the depression group. Although all three subgroups demonstrated some improvement in their emotional states compared to the initial stage, their progress showed signs of regression when compared to the middle stage. In the early stage, both the quarter-note and eighth-note rhythm subgroups showed slight improvements, while the sixteenth-note rhythm subgroup experienced a worsening of emotional states. Interestingly, after the early stage, the sixteenth-note subgroup showed a positive shift in emotional improvement, with the frequency of low-level emotional vocabulary rising from fewer than five times in the early stage to seven times in the middle stage. The eighth-note subgroup also showed a significant emotional shift, reaching a peak frequency of 10 times for low-level emotional vocabulary in the middle stage. In contrast, the quarter-note subgroup displayed a slight improvement in the early stage but then plateaued or even slightly declined in the middle stage, maintaining a frequency of fewer than five times. Unexpectedly, all three subgroups experienced a decline in emotional improvement during the later stage compared to the middle stage. The quarter-note subgroup showed the lowest level of emotional improvement, followed by the sixteenth-note subgroup. The eighth-note subgroup showed the greatest improvement. Among the three subgroups, the eighth-note subgroup demonstrated the best overall improvement in emotional state, while the quarter-note subgroup had the least improvement. The sixteenth-note subgroup exhibited an interesting pattern, worsening in the early stage, then improving significantly in the middle stage, before declining again in the later stage. Nevertheless, all three subgroups—quarter-note, eighth-note, and sixteenth-note—showed some improvement compared to the early stage, with frequencies of emotional vocabulary at 5, 9, and 6 times, respectively.

#### 5.4.3 Self-reflection report coding diagram for the stress imbalance group

**Figure 6:**
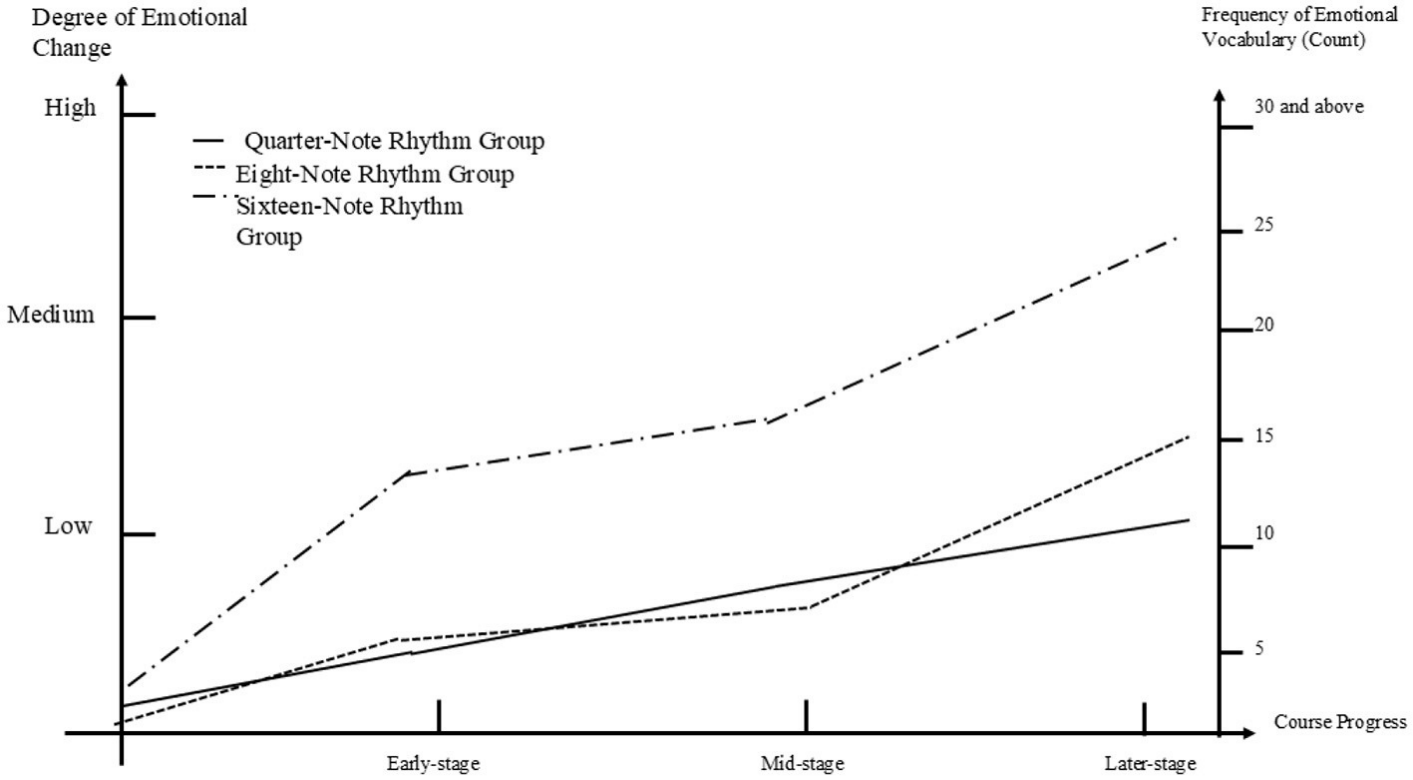
Coding for the pressure group.

Figure 6 illustrates the emotional transition process of participants in the three subgroups of the stress group. All three subgroups showed a positive trend in emotional changes. In the early stage, all three subgroups experienced improvement, with the sixteenth-note rhythm subgroup showing a rapid and significant increase, indicated by a frequency of 14 instances of moderate-level emotional vocabulary. Both the quarter-note and eighth-note rhythm subgroups also improved, but their levels were much lower compared to the sixteenth-note subgroup, with low-level emotional vocabulary used 5 times and 6 times, respectively. In the middle stage, both the sixteenth-note and quarter-note rhythm subgroups continued to improve, with the quarter-note subgroup surpassing the eighth-note subgroup, which showed no change during this period. In the later stage, all three subgroups demonstrated rapid improvement once again, with the sixteenth-note rhythm subgroup continuing to show the most significant progress. The eighth-note subgroup also improved considerably during this stage, while the quarter-note subgroup showed the least improvement compared to the other two subgroups. Among the three subgroups, the sixteenth-note rhythm subgroup showed the fastest and most pronounced improvement, with a final frequency of 25 instances of high-level emotional vocabulary. The quarter-note subgroup showed some improvement compared to earlier stages but remained the group with the least progress, with a final frequency of 12 instances of low-level emotional vocabulary. Notably, the eighth-note rhythm subgroup demonstrated a pattern of rapid improvement in both the early and later stages, but no change during the middle stage, ultimately ending with 15 instances of moderate-level emotional vocabulary.

#### 5.4.4 Comparison diagram of the most significant emotional transitions among the depression, anxiety, and stress imbalance groups

**Figure 7:**
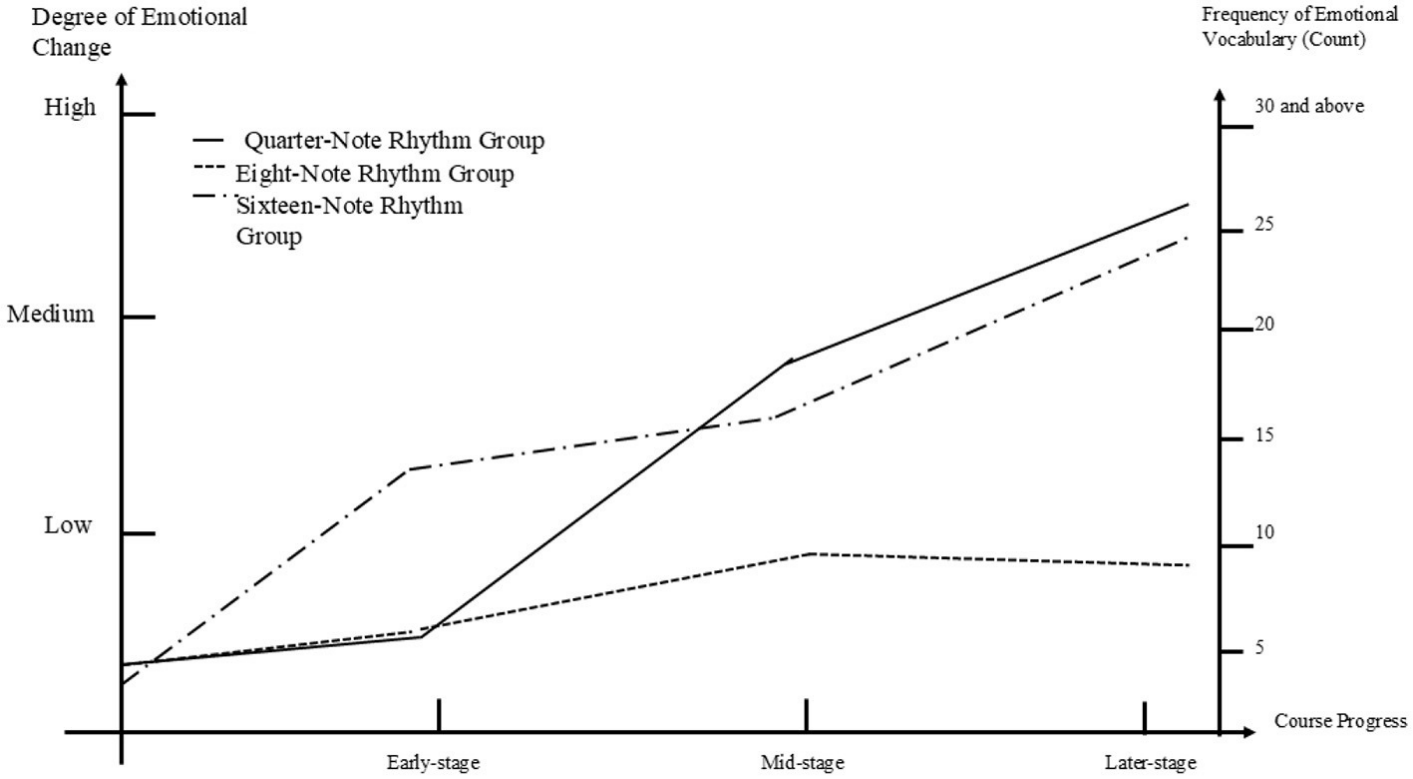
Comparison of the most significant emotional transitions among the depression, anxiety, and stress imbalance groups.

Figure 7 presents the coding chart of the most improved subgroups from the depression, anxiety, and stress imbalance groups. From the Figure 7, it can be observed that both the anxiety and stress groups showed significant improvement, whereas the depression group displayed more stable or even declining trends. In the early stage, all three groups experienced some improvement, with the stress group showing the most notable progress, while the anxiety and depression groups demonstrated only slight improvements. The middle stage marked a rapid increase for all three groups. Notably, the anxiety group improved the fastest, quickly becoming the group with the greatest level of improvement among the three group. Both the stress and depression groups also showed improvement during this stage, although at a more moderate pace. In the later stage, the stress and anxiety groups continued the positive trend observed in the middle stage, whereas the depression group showed a decline, in contrast to the other two groups. Overall, the anxiety group exhibited the most significant improvement, followed by the stress group, while the depression group showed the least improvement.

## 6 Discussion

The primary objective of this study is to investigate how the predominant method of integrating mindfulness with rhythmic music in the SEL program assists students in their emotional transitions and whether it provides effective instructional support for teachers. The main goals of this study are: (1) to examine whether the program can specifically alleviate emotional issues related to depression, anxiety, and stress imbalances in students, and if so, which type of rhythmic music should be used to address these issues; (2) to explore whether the degree of emotional alleviation has a differential impact on students' academic performance; and (3) to clarify the mechanisms behind the emotional transitions of students after participating in the program, identify the patterns involved, and assess whether the teaching support and deep interactions between teachers and students have improved. The following sections will discuss these results in greater detail.

### 6.1 Two-way analysis of variance data discussion

#### 6.1.1 Differences in emotional improvement among the anxiety, depression, and stress imbalance groups

The experimental results, derived from the comparison of pretest and posttest data, indicate that the mindfulness-based rhythmic music SEL program positively alleviates students' emotional issues related to depression, anxiety, and stress imbalances. Furthermore, different musical rhythms exhibit a preferential alleviation effect on these three types of emotional issues, as detailed below: Among the three subgroups within the anxiety group, the quarter-note rhythm subgroup showed the most significant reduction in anxiety levels when comparing pretest and posttest results (see Table 5), followed by the eighth-note rhythm subgroup and the sixteenth-note rhythm subgroup.

It is noteworthy that the findings for the pressure group were exactly the opposite of those for the anxiety group; the posttest results showed that the sixteenth-note rhythm subgroup experienced the most significant reduction in pressure levels, followed by the eighth-note rhythm subgroup and the quarter-note rhythm subgroup (see Table 7). This suggests that faster musical rhythms are more effective in alleviating pressure, while slower rhythms are less effective at significantly lowering pressure levels. This may be attributed to a significant correlation between the speed of musical rhythms and an individual's desire for movement. Specifically, when a person listens to music, changes in rhythm can activate areas of the brain related to movement, leading individuals to sway along with the musical rhythm. Such movements, like swaying, can help reduce stress and tension levels by regulating the release of cortisol, a stress hormone, in the brain (Gurusathya, 2019).

It is particularly noteworthy that for the depression group, music with a moderate tempo, such as the eighth-note rhythm, had the most significant positive effect in reducing depression levels. Extremely slow or overly fast rhythms were found to be less effective in alleviating depressive symptoms. Interestingly, faster music rhythms were found to have a greater alleviating effect on depression compared to slower rhythms. The results showed that the sixteenth-note rhythm subgroup exhibited more relief from depressive symptoms compared to the eighth-note rhythm subgroup. This result can be explained by the psychological characteristics of individuals with depression, who often experience sadness or negative emotions. While slow rhythms can help individuals relax, they also tend to induce sadness due to their elongated and mellow nature. Although fast-paced rhythms may not directly provide a calming effect, they can encourage physical movement, which helps individuals release negative emotions and thereby alleviate depressive symptoms. Based on these findings, the mindfulness-integrated music rhythm program can offer targeted instructional support for students with depression, anxiety, and stress imbalance. Specifically, eighth-note rhythms are recommended for alleviating depression, quarter-note rhythms for anxiety, and sixteenth-note rhythms for stress imbalance.

#### 6.1.2 Differences in academic performance among depression, anxiety, and stress imbalance groups

Through the comparison of pretest and posttest experimental results, it was found that participants in the anxiety, depression, and stress imbalance groups all showed improvements in academic performance, indicating that the alleviation of emotional issues has a positive influence on students' academic outcomes to a certain extent. Notably, the degree of alleviation of emotional problems is generally positively correlated with students' academic performance. Within the three groups, the improvements in academic performance among different subgroups were not consistent. In the anxiety group, the subgroup that experienced the greatest increase in scores was the quarter-note rhythm subgroup, which also exhibited the most significant reduction in anxiety levels. This was followed by the eighth-note rhythm subgroup and the sixteenth-note rhythm subgroup, in that order. In the pressure group, participants in the sixteenth-note rhythm subgroup had the most substantial improvement in scores, significantly exceeding those in the eighth-note and quarter-note rhythm subgroups, with no significant difference between the eighth and sixteenth-note groups. The depression group presented a unique case; although the overall academic performance of participants improved compared to before the experiment, there were no significant differences in scores among the subgroups. While the eighth-note rhythm subgroup was the most effective in alleviating depression levels, the scores of its participants did not significantly exceed those of participants in the quarter-note and sixteenth-note rhythm subgroups. This outcome can be attributed to the physiological and psychological characteristics associated with depression, such as decreased attention, diminished interest in learning, lack of motivation, and heightened feelings of frustration, which are significant factors impacting academic performance. The alleviation of depressive emotions does not immediately reduce the severity of these symptoms, and depression is a long-term emotional health issue that requires extended periods of intervention for relief. Therefore, the depression group may need more prolonged engagement in the program to effectively alleviate symptoms and achieve ideal academic results.

### 6.2 Discussion of self-reflection report coding figures

Coding Figures 4–6 thoroughly illustrate the similarities and differences in the emotional change processes among participants in different subgroups. A key point of concern for the anxiety group is that after the completion of the initial phase of the course, the emotional changes among participants in all three subgroups remained at a low transition stage, indicating a relatively passive situation. We found that, although participants in the quarter-note rhythm subgroup (hereafter referred to as the “quarter group”) showed slightly better emotional improvements compared to those in the eighth-note rhythm subgroup (hereafter referred to as the “eighth group”) and the sixteenth-note rhythm subgroup (hereafter referred to as the “sixteenth group”), the overall emotional transitions across all three subgroups were not satisfactory. According to the report content, most participants expressed difficulty in maintaining focus during the mindfulness breathing exercises due to an inability to concentrate. This situation may lead to a loss of enthusiasm for course learning, highlighting the urgent need for timely guidance from teachers. Encouragingly, after the mid-phase of the course, all three subgroups exhibited a turning point toward more positive emotional changes. This improvement is attributed to the participants' commitment to practicing during the mid-phase of the course. Notably, the quarter group continued to show better emotional improvements than the other two groups. Participants in the quarter group reported that the soothing rhythm significantly helped them relax and improve their concentration, which is a key reason for their better emotional transformation in the later stages.

The overall emotional improvement in the depression group was less than ideal, with a notable worsening of emotions in all three subgroups after the final stage of the course. Analysis of participants' reports revealed that the introduction of social activity units too early in the course created significant pressure for participants. Although they were able to follow the course requirements and complete the activities, the social interaction elements imposed an emotional burden. This outcome is related to the decreased sense of self-worth often experienced by individuals with depression. Therefore, it is advisable for individuals with depression to only be integrated into social activity learning units after a thorough assessment of their psychological state. Interestingly, while both the quarter-note and eighth-note rhythm subgroups showed slight emotional improvement after the early stage of the course, participants in the sixteenth-note rhythm subgroup experienced a deterioration in emotional state. This may be due to the highly energetic nature of the sixteenth-note rhythm. Participants in this subgroup often reported that the fast-paced rhythm was initially difficult to adjust to, which heightened their feelings of unease. Fortunately, as the course progressed, participants gradually adapted to the fast tempo, leading to a rapid improvement in emotional state. Notably, by the end of the middle stage, the sixteenth-note subgroup showed a significant positive shift in their emotional condition.

The overall emotional transformation effect in the pressure group was quite positive, particularly in the later phase of the course, where all three subgroups exhibited excellent emotional changes. This improvement can be attributed to the social activity learning component, which provided them with opportunities to communicate and share with others, significantly alleviating their psychological pressure. Comparative analysis among the three subgroups revealed that the sixteenth-note rhythm subgroup demonstrated a remarkable improvement in emotional states compared to the quarter-note and eighth-note rhythm subgroups. Notably, after the completion of the initial phase, the sixteenth-note subgroup's emotions shifted rapidly from low levels to high levels. Participants in the sixteenth-note subgroup reported that the upbeat music inspired them to engage in physical activities like exercising, dancing, and singing, with some participants actually participating in these activities, greatly reducing their feelings of stress. In contrast, the eighth-note subgroup experienced a more gradual change in emotional states after the mid-phase of the course; however, the reports did not prominently describe this situation. We hypothesize that this may be related to individual preferences. Although this musical rhythm initially alleviated their stress, its effectiveness diminished when the novelty wore off (as individual preference declined), leading to a significant reduction in its stress-relieving effects.

Additionally, the coding diagram 7 reveals significant differences in the emotional transition processes among the most notable subgroups within the depression, anxiety, and stress imbalance groups. We found that participants in the sixteenth-note rhythm subgroup of the pressure group were more receptive to the mindfulness-based rhythmic music course method, demonstrating a stronger level of emotional improvement compared to those in the anxiety and depression groups. At the beginning of the course, participants in the quarter-note rhythm subgroup of the anxiety group and the eighth-note rhythm subgroup of the depression group struggled to engage effectively due to difficulties with the exercises. Notably, as participants became more immersed in their learning, those in the anxiety quarter-note subgroup and the pressure sixteenth-note subgroup exhibited rapid and positive emotional transitions, with a trend of continuous improvement. During this phase, both groups reported engaging in more self-regulatory behaviors, such as inquiring about their specific emotions or thoughts. In contrast, the eighth-note rhythm subgroup in the depression group reported greater difficulty adapting to the course structure, particularly regarding the social learning module. This suggests that individuals with depression may benefit from complementary interventions, such as individual psychological counseling.

## 7 Conclusion

This study is the first to employ a social-emotional learning (SEL) program methodology that integrates different rhythmic types of music with mindfulness practices to address the challenges faced by students with emotional issues related to depression, anxiety, and stress imbalances in SEL courses. Compared to previous SEL methodologies, the approach combining mindfulness with rhythmic music provides a deeper and more precise understanding of the following research findings:

(1) The mindfulness-based rhythmic music SEL program has a significant effect on alleviating emotional issues in students with depression, anxiety, and stress imbalances. Specifically, eighth-note rhythm music, quarter-note rhythm music, and sixteenth-note rhythm music have substantial positive impacts on the respective groups of students suffering from depression, anxiety, and stress imbalances.
(2) Emotional improvement may be one of the primary factors influencing academic performance. In the anxiety and stress imbalance groups, the subgroups that showed the greatest improvement in academic performance were also those with the most significant emotional improvement. specifically, the quarter-note subgroup for anxiety and the sixteenth-note subgroup for stress imbalance. For the depression group, due to the lack of marked overall emotional improvement, no significant academic improvement was observed across subgroups.
(3) One of the most encouraging findings is the clear identification of the time points at which students with depression, anxiety, and stress imbalances encounter difficulties during the learning process, as well as the instructional support needed to help them navigate these challenges. In the anxiety group, it is especially important for teachers to focus on guiding and explaining mindfulness training during the early stages of the course (particularly in the first three classes). Providing students with more instructional support on how to concentrate can significantly influence their interest, perseverance, and learning outcomes in subsequent classes. For the depression group, the most suitable musical rhythms are the eighth-note and sixteenth-note rhythms, which convey a sense of positivity and energy. Teachers can replace relatively upbeat music tracks in future lessons. Additionally, the depression group requires long-term course support; engaging in social skills training is not suitable in the short term (at least for the first 10 classes), as it may exacerbate the psychological burden on individuals with depression. It is recommended that teachers incorporate one-on-one counseling to help this population regain their social skills. Students with stress imbalances respond particularly well to upbeat music, which can have an immediate and effective impact on helping them release stress. Furthermore, teachers should pay attention to the frequency of music changes during the mid-phase of the course (after eight classes) or guide students to listen to their preferred upbeat rhythms, which can effectively stimulate students' individual preferences and enhance instructional effectiveness. Additionally, it is recommended that teachers incorporate more listening activities and physical activities into the course, such as aerobic exercises, dancing, or walking, as these can effectively help students release psychological stress.

Our research findings reveal the specific types of instructional support needed by students with emotional issues, as well as the timing patterns of such support. This provides a comprehensive reference for SEL-related educational personnel and institutions, including teachers, schools, and community workers, to clearly understand and effectively manage students with emotional problems. It effectively aids educators in identifying the learning difficulties faced by students with emotional issues, allowing them to provide appropriate instructional support, thereby enhancing the quality of teacher-student interactions. Enhancing the Outcomes of Online SEL Programs. To a certain extent, significantly alleviates the prevalence of emotional problems among college students, promotes the development of social-emotional skills within this population, and ultimately fosters the formation of a sustainable society.

Finally, this study has certain limitations. As traditional higher education evolves from a linear structure to an open-loop system, creating diverse, multicultural, and intergenerational student populations, further refinement of the mindfulness-integrated music rhythm SEL program will require more in-depth research on different emotional issues across these varied groups.

## Data Availability

The original contributions presented in the study are included in the article/Supplementary material, further inquiries can be directed to the corresponding author.
