# Supplementary material for: Mindfulness's moderating role applied on online SEL education
Source: Front Psychol. 2024 Nov 19;15:1499357. doi: 10.3389/fpsyg.2024.1499357 (PMC11611557; doi:10.3389/fpsyg.2024.1499357)
Supplement: Supplementary file 1 [file Data_Sheet_1.ZIP › Raw data/Baker Anxiety Scale (BAI) for firefight profession student-ENGLISH.pdf]

Name:____ DOB:____________________________

Date:_________

Beck Anxiety Inventory (BAI)

Below is a list of common symptoms of anxiety. Please carefully read each item in the list. Indicate how much you have been bothered by that symptom during the past month OR since your last infusion, if you have started the ketamine infusion therapy. Circle one numnber in the corresponding space in the row next to each symptom.

1.

0	I do not feel numbness or tingling
1	I mildly feel numbness or tingling but it doesn’t bother me much
2	I moderately feel numbness or tingling and it wasn’t pleasant
3	I severely feel numbness or tingling and it bothered me a lot

2.

0	I do not experience feeling hot
1	I mildly experience feeling hot but it doesn’t bother me much
2	I moderately experience feeling hot and it wasn’t pleasant
3	I severely experience feeling hot and it bothered me a lot

3.

0	I do not feel wobbliness in legs
1	I mildly experience wobbliness in legs but it doesn’t bother me much
2	I moderately experience wobbliness in legs and it wasn’t pleasant at times
3	I severely experience wobbliness in legs and it bothered me a lot

4.

0	I am able to relax
1	I am mildly unable to relax but it doesn’t bother me much
2	I am moderately unable to relax and it wasn’t pleasant at times
3	I am severely unable to relax and it bothered me a lot

5.

0	I do not have a fear of the worst happening
1	I mildly have a fear of the worst happening but it doesn’t bother me much
2	I moderately have a fear of the worst and it is not pleasant at time
3	I severely have a fear of the worst and it bothers me a lot

6.

- 0 I do not feel dizziness or lightheadedness
- 1 I mildly feel dizziness or lightheadedness but it doesn’t bother me much
- 2 I moderately feel dizziness or lightheadedness and it doesn’t feel pleasant at times
- 3 I severely feel dizziness or lightheadedness and it bothers me a lot

7.

- 0 I do not feel my heart pounding/racing
- 1 I mildly feel my heart pounding/racing but it doesn’t bother me much
- 2 I moderately feel my heart pounding/racing and it doesn’t feel pleasant at times
- 3 I severely feel my heart pounding/racing and it bothers me a lot

8.

- 0 I do not feel unsteady
- 1 I mildly feel unsteady but it doesn’t bother me much
- 2 I moderately unsteady and it doesn’t feel pleasant at times
- 3 I severely feel unsteady and it bothers me a lot

9.

- 0 I do not feel terrified or afraid
- 1 I mildly feel terrified or afraid but it doesn’t bother me much
- 2 I moderately feel terrified or afraid and it doesn’t feel pleasant at times
- 3 I severely feel terrified or afraid and it bothers me a lot

10.

- 0 I do not feel nervous
- 1 I mildly feel nervous but it doesn’t bother me much
- 2 I moderately feel nervous and it doesn’t feel pleasant at times
- 3 I severely feel nervous and it bothers me a lot

11.

- 0 I do not have a feeling of choking
- 1 I mildly have a feeling of choking but it doesn’t bother me much
- 2 I moderately have a feeling of choking and it doesn’t feel pleasant at times
- 3 I severely have a feeling of choking and it bothers me a lot

12.

0 I do not experience hands trembling

1 I mildly experience hands trembling but it doesn’t bother me much

2 I moderately experience hands trembling and it doesn’t feel pleasant at times

3 I severely experience hands trembling and it bothers me a lot

13.

0 I do not feel shaky/unsteady

1 I mildly feel shaky/unsteady but it doesn’t bother me much

2 I moderately feel shaky/unsteady and it doesn’t feel pleasant at times

3 I severely feel shaky/unsteady and it bothers me a lot

14.

0 I do not have a fear of losing control

1 I mildly have a fear of losing control but it doesn’t bother me much

2 I moderately have a fear of losing control and it doesn’t feel pleasant at times

3 I severely have a fear of losing control and it bothers me a lot

15.

0 I do not have difficulty in breathing

1 I mildly have difficulty in breathing but it doesn’t bother me much

2 I moderately have difficulty in breathing and it doesn’t feel pleasant at times

3 I severely have difficulty in breathing and it bothers me a lot

16.

0 I do not have a fear of dying

1 I mildly have a fear of dying but it doesn’t bother me much

2 I moderately have a fear of dying and it doesn’t feel pleasant at times

3 I severely have a fear of dying and it bothers me a lot

17.

0 I do not feel scared

1 I mildly feel scared but it doesn’t bother me much

2 I moderately feel scared and it doesn’t feel pleasant at times

3 I severely feel scared and it bothers me a lot

18.

0 I do not experience indigestion

1 I mildly experience indigestion but it doesn’t bother me much

2 I moderately experience indigestion and it doesn’t feel pleasant at times

3 I severely experience indigestion and it bothers me a lot

19.

0 I do not feel faint/lightheaded

1 I mildly feel faint/lightheaded but it doesn’t bother me much

2 I moderately feel faint/lightheaded and it doesn’t feel pleasant at times

3 I severely feel faint/lightheaded and it bothers me a lot

20.

0 I do not have face flushed

1 I mildly have face flushed but it doesn’t bother me much

2 I moderately have face flushed and it doesn’t feel pleasant at times

3 I severely have face flushed and it bothers me a lot

21.

0 I do not have hot/cold sweats

1 I mildly have hot/cold sweats but it doesn’t bother me much

2 I moderately hot/cold sweats and it doesn’t feel pleasant at times

3 I severely have hot/cold sweats and it bothers me a lot

The total score is calculated by finding the sum of the 21 items.

Beck Anxiety Score _______________

Score of 0 – 21 = low anxiety

Score of 22 – 35 = moderate anxiety

Score of 36 and above = potentially concerning levels of anxiety
