# Supplementary material for: Mindfulness's moderating role applied on online SEL education
Source: Front Psychol. 2024 Nov 19;15:1499357. doi: 10.3389/fpsyg.2024.1499357 (PMC11611557; doi:10.3389/fpsyg.2024.1499357)
Supplement: Supplementary file 1 [file Data_Sheet_1.ZIP › Raw data/Fire students perceived Stress Scale (PSS) test-ENGLISH.pdf]

Fire students perceived Stress Scale (PSS) test

Dear interviewee: Hello

Thank you for taking your time to participate in our survey! Your opinion is important to our project. This survey aims to understand the mental health of fire students, and we sincerely look forward to your truthful responses. In order to protect your privacy, we promise that this survey will be anonymous and your personal information will be kept strictly confidential. The questionnaire consists of 9 questions, please take into account your physical/psychological situation in the last week to answer. Although some of the questions look similar, they are actually different, so each question needs to be answered. Try to answer them quickly and unthinkingly, without thinking about the meaning behind each score calculation, so as to truly reflect your true stress perception. The entire questionnaire is expected to take about 5 minutes, so please complete it at your convenience. Your participation is important for us to gain a deeper understanding of the mental health issues of fire students. If you have any questions or need further information while completing the questionnaire, felt free to contact us and your feedback will be highly valued. Thank you again for your participation and look forward to hearing your valuable input!

Thank you very much.

1. Feeling upset that something unexpected has happened

0 Never 1 occasionally 2 sometimes 3 often 4 always

2. Feeling like you have no control over what's important in your life

0 Never 1 occasionally 2 sometimes 3 often 4 always

3. Feel jittery and stressed

0 never 1 occasionally 2 sometimes 3 often 4 always

4. Successfully deal with annoying life hassles

0 Never 1 occasionally 2 sometimes 3 often 4 always

5. Feel that you are dealing effectively with the important changes that are taking place in your life

0 Never 1 occasionally 2 sometimes 3 often 4 always

6. 4. Feel confident in your ability to handle your personal problems

0 Never 1 occasionally 2 sometimes 3 often 4 always

7. Feel that things are going well

0 Never 1 sometimes 2 sometimes 3 often 4 always

8. Find yourself unable to handle all the things you have to do

0 never 1 occasionally 2 sometimes 3 often 4 always9. There are ways to control the annoying things in your life

0 Never 1 occasionally 2 sometimes 3 often 4 always

10. Always feel that you are the master of things

0 Never 1 occasionally 2 sometimes 3 often 4 always

11. Always get angry because things happen that are out of your control

0 Never 1 occasionally 2 sometimes 3 often 4 always

12. Always think of something you have to do

0 Never 1 occasionally 2 sometimes 3 often 4 always

13. Always know how to schedule your time

0 Never 1 occasionally 2 sometimes 3 often 4 always

14. You often feel like you have a mountain of problems and can't overcome them
0 Never 1 occasionally 2 sometimes 3 often 4 always
