# Supplementary material for: Mindfulness's moderating role applied on online SEL education
Source: Front Psychol. 2024 Nov 19;15:1499357. doi: 10.3389/fpsyg.2024.1499357 (PMC11611557; doi:10.3389/fpsyg.2024.1499357)
Supplement: Supplementary file 1 [file Data_Sheet_1.ZIP › Raw data/Firefighting profession Student Depression (CES-D) test scale-ENGLISH.pdf]

Firefighting profession Student Depression (CES-D) test scale

Dear interviewee: Hello

Thank you for taking your time to participate in our survey! Your opinion is important to our project. This survey aims to understand the mental health of fire students, and we sincerely look forward to your truthful responses. In order to protect your privacy, we promise that this survey will be anonymous and your personal information will be kept strictly confidential. The questionnaire consists of 9 questions, please take into account your physical/psychological situation in the last week to answer. Although some of the questions look similar, they are actually different, so each question needs to be answered. Try to answer them quickly and unthinkingly, without thinking about the meaning behind each score calculation, so as to truly reflect your true stress perception. The entire questionnaire is expected to take about 5 minutes, so please complete it at your convenience. Your participation is important for us to gain a deeper understanding of the mental health issues of fire students. If you have any questions or need further information while completing the questionnaire, felt free to contact us and your feedback will be highly valued. Thank you again for your participation and look forward to hearing your valuable input!

Thank you very much.

1. Did you find yourself in a good mood this past week?

① No ② rarely ③ sometimes ④ often

2. Have you felt lonely in the past week?

① Never ② rarely ③ sometimes ④ often

3. Did you felt sad in the past week?

① No ② seldom ③ sometimes ④ often

4. Do you think your life has been good in the past week?

① Never ② rarely ③ sometimes ④ often

5. Did you felt like eating for the past week?

① No ② rarely ③ sometimes ④ often

6. Did you sleep badly in the past week?

① No ② rarely ③ sometimes ④ often

7. Did you felt worthless in the past week?

① Never ② rarely ③ sometimes ④ often

8. Did you felt like you had nothing to do in the past week?

① No ② rarely ③ sometimes ④ often

9. Did you find a lot of fun (interesting things) in your life this past week?

① Not ② seldom ③ sometimes ④ often
