# Supplementary material for: Mindfulness's moderating role applied on online SEL education
Source: Front Psychol. 2024 Nov 19;15:1499357. doi: 10.3389/fpsyg.2024.1499357 (PMC11611557; doi:10.3389/fpsyg.2024.1499357)
Supplement: Supplementary file 1 [file Data_Sheet_1.ZIP › Raw data/Firefighting profession students theory test-ENGLISH.pdf]

Firefighting profession students theory test

1. Choice question

1. When fighting stack fires, do not easily climb to the top of the burning stack, () on the top of the stack of barreled items, the shelf deformation or directly baked by the fire, should keep a sufficient safe distance.
A, should not B, should not C, should not D, strictly prohibited
Answer: D
2. When carrying out traffic road fire fighting or emergency rescue, the rescue vehicle should be stopped diagonically across the road (tail close to the direction of the car) behind the accident vehicle, and the parking position should be no less than () meters away from the accident point.
A, 15B, 25C, 30D, 50
Answer: D
3. The transfer tank must be operated by professionals. When the transfer tank of flammable and explosive substances is implemented, the pipeline and equipment must be (). It is strictly prohibited to drag the transfer tank equipment on the ground.
A, fixed B, cooling C, grounding D, strapping
Answer: C
4. The vehicle should control the speed while driving, and maintain a sufficient safe distance from the front car, strictly prohibited (), to prevent motorcycles, bicycles, pedestrians, vehicles across and rapid turns and lane changes.
A, forced overtaking B, following the car C, honking D, horn Shouting
Answer: A
5. In the geological disaster scene rescue, to set up observation posts, near the () mountain road side. And formulate an emergency evacuation plan in advance, clear the evacuation route, unified evacuation signals, and strictly prevent casualties caused by secondary disasters.
A, close to B, far away from C, close to D, adjacent
Answer: B
6. On-site warning must be based on the category, characteristics, scale and scope of the disaster, scientifically determine the warning area, delimit the warning scope and regional division, and strictly prohibit () entering.
A, unrelated vehicles B, unrelated personnel and vehicles C, media reporters D, onlookers
Answer: B
7. How often does the detachment hold a security situation analysis meeting ()?
A, every six months B, every quarter C, every two months D, monthly
Answer: D
8. Fire rescue stations must be set up () to carry out special training on search and rescue of commanders and combatants in distress (demolition, search, rescue, transfer and escape self-rescue, etc.) to improve emergency rescue capabilities.
A, safety inspection team B, assault group C, emergency rescue team D, demolition search and rescue team
Answer: C
9. The breathing mask should be fitted with my face shape, dedicated, () maintenance. (1

point)
A, Unified B, alone C, centralized D, self
Answer: D
10. Cavitation lighting must be implemented by () and with the cooperation of engineering and technical personnel, it must be approached from the upwind or side upwind direction, and maintain a sufficient safe distance.
A, expert argumentation B, superior instruction C, reasonable speculation D, environmental assessment
Answer: A

2. Multiple choice (total questions)

1. When evacuating flammable, explosive and corrosive items, you should (). (1 point)
A, classification place B, draw a warning line C, set up the corresponding warning sign D, arrange special care E, prohibit irrelevant personnel near
Answer: ABCDE
2. When entering the area of high temperature and humidity, strong thermal radiation and explosion risk, fire insulation clothing, fire protection clothing or explosion-proof clothing should be worn ().
A, anti-static underwear B, anti-static gloves, socks C, anti-high temperature gloves D, cooling vest
Answer: CD
3. Alert personnel should dress in a standard manner, bring all the equipment such as (), stick to their posts, observe closely, report and issue warning signals in time when an emergency is found, and quickly organize evacuation. (1 point)
A, Communication B, lighting C, Warning D, warning
Answer: ABCD
4. Before the drill, it is necessary to conduct a comprehensive inspection of the safety of the drill site and the () used for the drill.
A, building, B, device, C, vehicle D, personnel
Answer: ABC
5. People who enter the dangerous area of leakage should wear () to avoid contact with live bodies;
A, insulation clothing B, insulation gloves C, insulation rubber boots D, fire protection clothing
Answer: ABC
6. When wading in water, pay attention to avoid live equipment and lines as well as trenches, manholes, caves and other dangerous parts under the water surface. When approaching buildings, be alert to falling objects from high heights and unstable collapse of components to prevent dangerous situations.
A, electric shock B, fall C, suction D, hit
Answer: ABCD
7. When floor fighting, the water distributor position is usually set at the main entrance, and the burning layer higher than 6 floors is generally set in the fire layer () or () stairwell.
A, the next floor B, the next two floors C, the upper floor D, the upper two
floors
Answer: AB

8. In () and other meteorological conditions, or through snow and ice, mud, slippery and other roads, should slow down, turn on fog lights, danger warning lights, and install anti-skid chains if necessary.
A, strong wind B, rain C, snow D, fog
Answer: ABCD
9. Usually the gunner and the coordination personnel to () emergency hedging and rapid evacuation.
A, keep a distance from B, cross standing C, easy to observe D, cooperate
Answer: ABC
10. When teammates () and other situations occur, should immediately take cardiopulmonary resuscitation and other emergency measures, must be continuous, and uninterrupted.
A, poisoning B, electric shock C, drowning D, suffocation
Answer: BCD
3. True or false (total question)
1. In a live environment, when the reconnaissance is moving forward, the hand should be turned inward, and the bare wire should not be backed against the wall to prevent the insulation layer of the wire from burning and aging and causing electric shock.
A, correct B, wrong
Answer: B
2. When dealing with highway tunnel disasters, vehicles should generally be parked on the left outside the holes at both ends of the tunnel.
A, correct B, wrong
Answer: B
3. Before entering inflammable and explosive and other high-risk places, the vehicle must be installed with fire cover and parked in the upwind or downwind position. (1 point)
A, correct B, wrong
Answer: B
4. When implementing smoke exhaust and poison reduction, fully consider the flow direction of smoke and gas, and reasonably choose the air intake, smoke exhaust and dispersion direction. At the site of toxic and harmful substances burning or leaking accidents, it is necessary to carry out uninterrupted reconnaissance and detection throughout the whole process, and reduce the concentration of toxic and harmful substances at the site by dilution, dispersion, neutralization and other methods.
A, correct B, wrong
Answer: A
5. Enter flammable and explosive areas, should wear fire protective clothing, explosion-proof clothing and other special protective equipment.
A, correct B, wrong
Answer: B
6. Every half year, detachment every quarter, brigade and station every month to carry out at least 1 fire rescue combat training safety situation analysis, find responsibility implementation, personnel allocation, learning and education, business training, combat operations, equipment maintenance, vehicle inspection, traffic management and other aspects of the problems, analyze the reasons, summarize lessons, formulate targeted improvement

measures. To participate in major fire fighting and rescue operations and carry out high-risk training, safety risk assessment should be organized and necessary safety precautions should be taken.
A) Correct B) Wrong
Answer: A
7. The valve plugging group is generally 2 to 3 people, the personnel are lean, should be carried out under the guidance of the process disposal team or the technical staff of the factory, if necessary, the standby force is organized for rotation, and the emergency rescue team is on standby in the designated position.
A, correct B, wrong
Answer: A
8. When the vehicle is driving, the driver should strengthen traffic observation, and the driver should not pick up the phone and fatigue driving;
A, correct B, wrong
Answer: B
9. When carrying out fire fighting and rescue on traffic roads, rescue vehicles should, in principle, stop at a position not less than 50 meters away from the accident point, and stop diagonically across the road (the rear of the vehicle is close to the direction of the coming vehicle).
A, correct B, wrong
Answer: A
10. Physical training must follow the rules of movement, adhere to the principle of step by step, scientific organization, should not rush to success.
A, right B, wrong
Answer: A
